# Cell-Type-Specific Gene Expression in Developing Mouse Neocortex: Intermediate Progenitors Implicated in Axon Development

**DOI:** 10.3389/fnmol.2021.686034

**Published:** 2021-07-12

**Authors:** Francesco Bedogni, Robert F. Hevner

**Affiliations:** ^1^School of Biosciences, Cardiff University, Cardiff, United Kingdom; ^2^Department of Pathology, University of California, San Diego, La Jolla, CA, United States

**Keywords:** radial glia, intermediate progenitors, epithelial-mesenchymal transition, planar cell polarity, Wnt-PCP, cortical development, touch-and-go

## Abstract

Cerebral cortex projection neurons (PNs) are generated from intermediate progenitors (IPs), which are in turn derived from radial glial progenitors (RGPs). To investigate developmental processes in IPs, we profiled IP transcriptomes in embryonic mouse neocortex, using transgenic *Tbr2*-GFP mice, cell sorting, and microarrays. These data were used in combination with *in situ* hybridization to ascertain gene sets specific for IPs, RGPs, PNs, interneurons, and other neural and non-neural cell types. RGP-selective transcripts (*n* = 419) included molecules for Notch receptor signaling, proliferation, neural stem cell identity, apical junctions, necroptosis, hippo pathway, and NF-κB pathway. RGPs also expressed specific genes for critical interactions with meningeal and vascular cells. In contrast, IP-selective genes (*n* = 136) encoded molecules for activated Delta ligand presentation, epithelial-mesenchymal transition, core planar cell polarity (PCP), axon genesis, and intrinsic excitability. Interestingly, IPs expressed several “dependence receptors” (*Unc5d*, *Dcc*, *Ntrk3*, and *Epha4*) that induce apoptosis in the absence of ligand, suggesting a competitive mechanism for IPs and new PNs to detect key environmental cues or die. Overall, our results imply a novel role for IPs in the patterning of neuronal polarization, axon differentiation, and intrinsic excitability prior to mitosis. Significantly, IPs highly express Wnt-PCP, netrin, and semaphorin pathway molecules known to regulate axon polarization in other systems. In sum, IPs not only amplify neurogenesis quantitatively, but also molecularly “prime” new PNs for axogenesis, guidance, and excitability.

## Introduction

Intermediate progenitors (IPs) are a type of cortical progenitors “intermediate” in the lineage from radial glial progenitors (RGPs), which produce IPs, and projection neurons (PNs), which are generated from IPs (Haubensak et al., 2004; Miyata et al., 2004; Noctor et al., 2004). RGPs have high self-renewal capacity and multilineage differentiation potential, and are thus considered to be a class of neural stem cells (NSCs) (Taverna et al., 2014). In contrast, IPs have low proliferative capacity and single lineage commitment to produce only glutamatergic PNs, and thus are neural progenitor cells but not NSCs (Mihalas and Hevner, 2018). In mice, RGPs and IPs are further distinguished by morphology, expression of transcription factors (TFs) such as Sox9 and Tbr2 (respectively); and by cell body location in the ventricular zone (VZ) for RGPs, or VZ and subventricular zone (SVZ) for IPs (Kowalczyk et al., 2009; Hevner, 2019). In mice, IPs generate the vast majority (possibly all) of the PNs in all cortical layers, including Cajal-Retzius cells and subplate neurons (Haubensak et al., 2004; Kowalczyk et al., 2009; Mihalas et al., 2016).

Cortical IPs are a unique cell type in vertebrate neurogenesis, but their significance in development remains uncertain. One proposed advantage of IPs is that they can divide away from the ventricular surface, to reduce crowding and increase neurogenic output per VZ surface area (Taverna et al., 2014). Also, IPs play a crucial role in Delta-Notch signaling as the major source of Delta-like signals that activate Notch and prevent premature RGP differentiation (Yoon et al., 2008; Nelson et al., 2013). In addition, IPs interact with migrating interneurons (INs) by secreting chemokine *Cxcl12* (SDF-1), which binds to *Cxcr4* and *Ackr3* receptors on INs to guide their tangential migration (Sessa et al., 2010; Saaber et al., 2019). Previously, it was suggested that IPs are specialized to amplify upper layer neurons; however, IPs were found to produce the majority of PNs in lower as well as upper layers (reviewed by Hevner, 2019). Evolutionarily, IPs are thought to serve as a cellular substrate for development of gyral folds (Kriegstein et al., 2006; Hevner, 2016; Toda et al., 2016).

In the present study, we hypothesized that IPs may play additional, unknown roles in cortical development, which might be revealed by transcriptome analysis. Our goals were: (1) to identify genes that are selectively expressed in IPs; (2) to analyze the pathways of IP-specific genes, using context-specific annotations from previous studies of neocortex; and (3) to identify developmental processes that are selectively activated in IPs, and compare them to those in RGPs and PNs. As part of this analysis, we ascertained gene sets for other cell types and features of E14.5 mouse neocortex, including neocortex-specific properties such as rostrocaudal patterning and PN laminar fate.

Previous studies of mouse IP transcriptomes, using different approaches, have produced distinct perspectives. An early single-cell transcriptome study using microarrays and unbiased cluster analysis distinguished RGPs, two types of IPs, and new PNs as cell types in the embryonic mouse VZ and SVZ (Kawaguchi et al., 2008). That study divided IPs into “type II” or apical IPs (aIPs), and “type III” or basal IPs (bIPs). (“Type I” cells were RGPs, and “type IV” were new PNs). The aIPs and bIPs were found to share expression of many genes, including *Tbr2* (MGI: *Eomes*), but also exhibited some transcriptome differences. That transcriptome study accorded with histological results showing that Tbr2+ IPs occupy distinct bands in the VZ and SVZ (Englund et al., 2005; Kowalczyk et al., 2009). Subsequently, a different study used cells sorted from *Tbr2*-GFP mouse neocortex to compare IP gene expression across embryonic ages (Cameron et al., 2012); however, due to the study design, general markers of IPs (such as *Tbr2*) were not ascertained. More recently, single-cell analyses of temporally defined RGP-IP-PN lineages reported transcriptional waves associated with PN differentiation (Telley et al., 2016, 2019). However, those single-cell results were not all validated by *in situ* hybridization (ISH), and we have found that many putative markers of RGPs, IPs, and PNs from that study do not show expected patterns on ISH. For example, some proposed IP markers (such as *Dbt*, *Pfkm*, and *Rprm*) show strong expression in the CP on ISH, consistent with postmitotic PNs. In the present study, we hypothesized that a new approach and analysis of IP transcriptomes could improve our knowledge of IP-selective genes and developmental mechanisms.

To profile IP gene expression, we sorted GFP+ cells and GFP− cells from embryonic day (E) 14.5 *Tbr2*-GFP mouse neocortex, then compared their transcriptomes using microarrays. Partial analyses of these data have been published previously (Bedogni et al., 2010; Nelson et al., 2013; Elsen et al., 2018), but this is the first comprehensive analysis to ascertain cell-type-specific gene sets. Genes (transcripts) that were highly enriched in either GFP+ cells or GFP− cells were further evaluated by ISH and literature search. Using known correlations between histological zone, gene expression, and cell identity in embryonic neocortex (Kawaguchi et al., 2008; Ayoub et al., 2011), we determined that gene expression was cell-type-selective only if microarray and ISH criteria met specific criteria (see section “Materials and Methods”). This approach enabled us to identify gene sets for all known cell types (neural and non-neural) in E14.5 mouse neocortex.

Non-neural cell types in developing neocortex are known to include microglia, leptomeninges, vascular, and blood cells. The vascular and blood elements are each further divided into multiple types: endothelium and pericytes for vascular; erythrocytes, monocytes, T lymphocytes, and B lymphocytes for blood. The gene sets for these cell types were useful to evaluate interactions between neural and non-neural cells during cortical development, such as those between RGPs and meningeal cells (Myshrall et al., 2012; Dasgupta and Jeong, 2019), and between RGPs and blood vessels (Biswas et al., 2020).

Our results demonstrate: (1) that each cell type in E14.5 mouse neocortex is characterized by the expression of specific gene sets; (2) that different cell types activate distinct signaling pathways; (3) that IPs likely play previously unsuspected roles in defining neuronal polarity and axogenesis; and (4) that extensive interactions occur between diverse cell types to coordinately regulate the growth, organization, and homeostasis of this complex brain region.

## Results

### Cell-Type-Specific Gene Expression Determined by Microarray and *in situ* Hybridization

**FIGURE 1:**
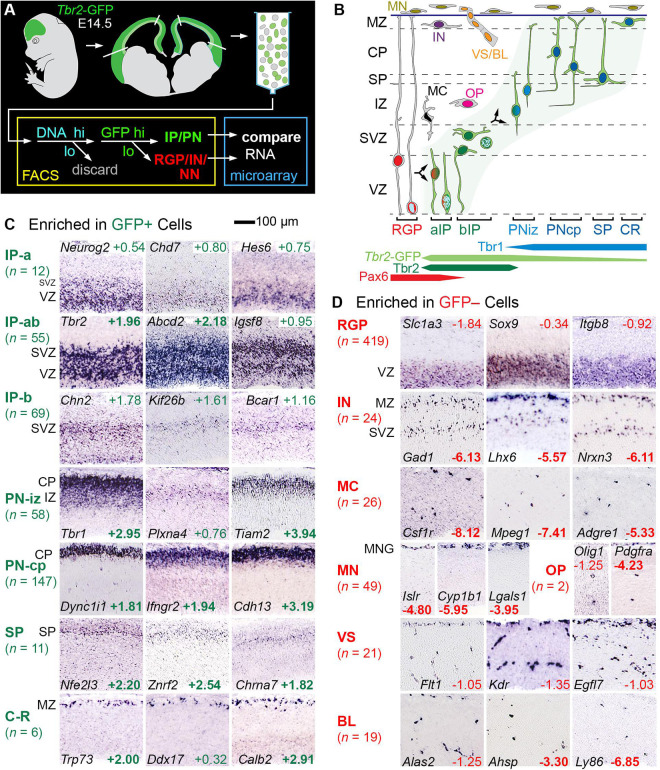
Determination of cell type-selective gene expression in E14.5 mouse neocortex. **(A)** Cells from E14.5 *Tbr2*-GFP neocortex were sorted into GFP+ and GFP– cells, each profiled with transcriptome microarrays. Genes enriched in either group were studied by ISH. Note that sorting by DNA content evidently did not exclude postmitotic cells (see test for details). **(B)** Schematic of cell types and TF expression in E14.5 neocortex. **(C)** GFP+ cell types included IPs and PNs in various stages of differentiation, as indicated by typical ISH patterns. Some IP genes were expressed in the VZ and SVZ, indicating apical and basal IPs (IP-ab); others selectively in VZ or SVZ, indicating enrichment in aIP or bIP cells, respectively. **(D)** GFP– cell types showed characteristic ISH patterns. Numbers are log_2_FC values, in colored text if significant (*p* < 0.05), and bold if in the top 300 positively or negatively enriched genes. ISH: Genepaint. Abbreviations: see text.

To identify genes expressed selectively by IPs and other cell types, we correlated microarray transcriptome profiling of lineage-sorted cells with ISH expression patterns in embryonic neocortex (Figure 1). *Tbr2*-GFP neocortex (E14.5) was dissociated, and cells were sorted into GFP+ and GFP− bins, after first enriching for progenitor cells on the basis of DNA content (Figure 1A). Importantly, despite sorting by DNA content, postmitotic cells were evidently not excluded, as they were well represented in the transcriptome results; the most likely explanation is that cell dissociation was incomplete, with doublets sorted as high-DNA cells.

RNA was amplified from GFP+ and GFP− cells for hybridization on Affymetrix Mouse Gene 430 2.0 microarrays (Nelson et al., 2013). In this experiment, GFP+ cells were significantly enriched (unadjusted *p* < 0.05) in 4,685 genes, and GFP− cells were significantly enriched in 3,262 genes. Gene enrichment was expressed quantitatively as log_2_ of the fold change (log_2_FC), which was positive for genes enriched in GFP+ cells, and negative for genes enriched in GFP− cells. The raw microarray results are presented in Supplementary Table 1. Consolidated microarray results are presented along with other information about each gene (ISH expression pattern, literature references, etc.) in Supplementary Table 2 (the primary integrated resource for this paper). Gene sets for all of the cell types and other features ascertained here are presented in Supplementary Tables 3–10.

Since GFP fluorescence in *Tbr2*-GFP neocortex labels not only IPs, but also postmitotic PNs (due to passive GFP inheritance), GFP+ cells encompassed the entire IP-PN lineage, including Cajal-Retzius and subplate neurons. Conversely, GFP– cells included RGPs and other non-*Tbr2*-expressing cell types, including all non-neural lineages. We evaluated the top 300 enriched genes in GFP+ cells and GFP− cells by ISH, using online databases and previous studies as described (Bedogni et al., 2010). Genes that met both criteria of (1) enrichment in GFP+ cells or GFP− cells, and (2) characteristic ISH expression patterns, were ascertained as cell-type-selective (Figure 1 and Supplementary Table 2 column I). (Genes are called “cell-type-selective” rather than “cell-type-specific” because very few genes are truly specific for one cell type).

Thus, cell-type-selectivity was strictly defined by criteria from both microarray and ISH data (Figures 1C,D; see section “Materials and Methods” for details). For example, IP-selective genes were (1) significantly enriched in GFP+ cells, and (2) expressed mainly in VZ (aIPs), VZ and SVZ (all IPs), or SVZ (bIPs) (Figure 1C). Genes that were expressed in all IPs, such as *Tbr2*, were classified as IP-ab genes (*n* = 55). Genes expressed mainly in aIPs were designated IP-a (*n* = 12), and genes mainly in bIP cells were designated IP-b (*n* = 69). Thus, genes expressed in aIPs included IP-a and IP-ab genes, while genes expressed in bIPs included IP-b and IP-ab genes. The standard for determining localization in VZ and SVZ was *Tbr2* (Figure 1C). Detailed analyses of IP- and RGP-selective are given below, following brief descriptions of additional features captured in our analysis.

### Pallial Identity, Rostrocaudal Patterning, and Laminar/Axonal Projection Subtypes

**FIGURE 2:**
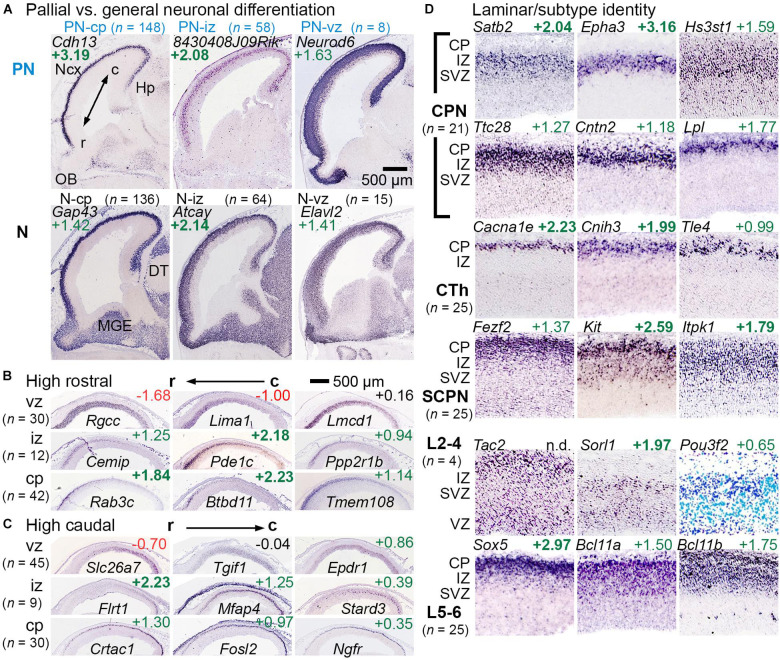
Gene sets for regional, laminar, and axonal projection identity or fate. **(A)** Genes that were expressed mainly in pallium but not ventral forebrain were categorized as PN genes, while genes expressed widely expressed in differentiation zones were categorized as general neuronal (N). PN and N genes were found in each histologic zone, categorized according to the deepest zone of expression. Not shown: PN-svz (*n* = 14) and N-svz (*n* = 48). R-C indicates the rostrocaudal axis. **(B,C)** Rostrocaudal expression gradients were typically found within 1–2 contiguous zones. **(D)** Genes linked to specific laminar and axonal projection fates were categorized (see section “Materials and Methods”). Abbreviations: CPN, callosal projection neuron; CTh, corticothalamic; SCPN, subcerebral projection neuron. n.d., not detected.

Among genes enriched in GFP+ cells (IP-PN lineage), some were expressed predominantly in the pallium (cortical primordium) on ISH, with little expression in subpallial forebrain. These pallial-selective genes, which may be important in cortex-specific differentiation, were designated PN-cp, PN-iz, PN-svz, or PN-vz, according to zonal expression patterns (Figure 2A, upper row “PN”). Other genes enriched in GFP+ cells were more broadly expressed in pallial and subpallial differentiation zones on ISH. These genes, representing general neuronal differentiation, were designated N-cp, N-iz, N-svz, or N-vz (Figure 2A, lower row “N”). Interestingly, many markers of PN or general neuronal differentiation, such as *Elavl2*, were initially expressed in the VZ of cortex and were maintained in more superficial zones, suggesting they are initially activated in aIPs (Figure 2A). Gene sets for the IP-PN lineage are listed in Supplementary Table 3, and for general neuronal differentiation in Supplementary Table 4.

Rostrocaudal expression gradients were noted for some genes (Figures 2B,C). For most such genes, expression was confined within one or two adjacent zones (VZ, SVZ, IZ, and CP) rather than spanning multiple zones (Elsen et al., 2013). Markers of the cortical hem and antihem, which serve as cortical patterning centers (Subramanian et al., 2009), were also noted (Supplementary Figure 1), as were some genes with hippocampal-restricted expression. Gene sets for rostrocaudal markers, hem, antihem, and hippocampus are given in Supplementary Table 5.

Laminar fates and axonal projection-defined subtypes of PNs could not be inferred directly from gene expression data, but candidate gene sets for these features were assembled from previous studies (see section “Materials and Methods”). Genes were confirmed as PN laminar fate or axon target markers if they were enriched in GFP+ cells, and exhibited zonal expression consistent with the proposed identity (Figure 2D). For example, early-born corticothalamic (CTh) PNs are expected to reside in the cortical plate on E14.5, while late-born upper layers 2-4 (L2-4) neuron precursors are expected mainly in the SVZ and IZ. Since callosal projection neurons (CPNs) are found in all neocortical layers (although enriched in upper layers), CPN-selective genes could be expressed in any zone. Gene sets for PN laminar fate and axonal target identity are presented in Supplementary Table 6.

### Proliferation Markers and Neural Stem Cell Identity

Transcriptional markers of proliferative activity have been established in previous studies (Whitfield et al., 2006). These were screened against our microarray and ISH results (Supplementary Figure 2A). The vast majority of proliferation markers were enriched in GFP− cells, and were localized in the VZ on ISH, thus satisfying criteria for RGP-selective genes. Additional markers of proliferative activity were ascertained by annotating known cell cycle functions. In contrast to proliferation genes, molecules linked to cell cycle exit or quiescence were enriched in GFP+ cells, and exhibited various expression patterns on ISH (Supplementary Figure 2B). Gene sets for proliferation and cell cycle exit are listed in Supplementary Table 7.

Proposed markers of neural stem cell (NSC) and progenitor (NSPC) identity (Easterday et al., 2003; Andreotti et al., 2019) were screened in the context of developing neocortex, using our microarray and ISH data. Since RGPs exhibit properties of NSCs while IPs do not (Taverna et al., 2014; Hevner, 2019), candidate NSC marker genes were confirmed only if RGP-selective (*n* = 15; Supplementary Figure 2C). Some candidate NSC or NSPC genes were expressed in the VZ/SVZ, but were not RGP-selective by microarray criteria. Such genes (*n* = 9; Supplementary Figure 2D) were identified as markers of neural stem and progenitor cells (NSPCs), likely expressed in both RGPs and IPs. Indeed, this expression pattern has been reported for *Nes* (Mignone et al., 2004) and *Msi1* (Kawase et al., 2011). Gene sets for cortical NSCs and NSPCs are listed in Supplementary Table 7. Since Notch signaling is critical for NSC maintenance, genes for Notch signaling are also listed in Supplementary Table 7 (and are further discussed below).

### Radial Glial Progenitor Identity

#### RGP-Selective Transcripts

**FIGURE 3:**
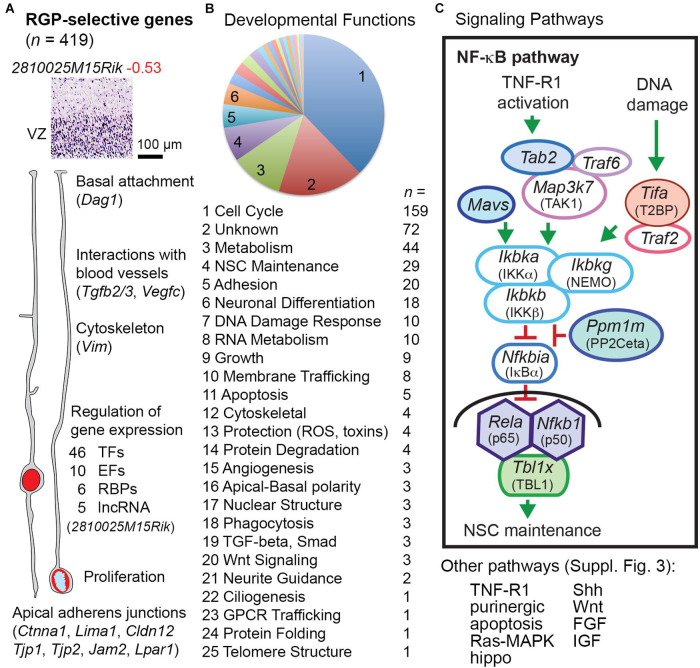
RGP-selective genes. **(A)** The example transcript (*2810025M15Rik*) is a ncRNA of unknown function. Some key functions of RGP-selective genes and examples are indicated. **(B)** Developmental function categories of RGP-selective genes. **(C)** RGP-selective genes (indicated by colored shading) were highly linked to certain signaling pathways such as NF-κB. Components of the transcription complex (p65, p50, and TBL1) were all RGP-selective, as were upstream activators and repressors of the pathway. Shapes with white fill represent genes expressed in multiple cell types.

Radial glial progenitor-selective genes (*n* = 419; Supplementary Table 8) were significantly enriched in GFP− cells, and were expressed mainly in the VZ (Figures 1D, 3). Our analysis confirmed classic markers of RG identity including *Slc1a3* (GLAST), *Notch1*, *Glul* (glutamine synthetase), *Sox9*, and *Vim* (vimentin) (Supplementary Table 2 column I). Many RGP-selective genes were devoted to gene regulation, including 46 TFs, 10 non-TF epigenetic factors (Elsen et al., 2018), 6 RNA-binding proteins (RBPs), and 5 lncRNAs, for a total of 67 RGP-selective regulators of gene expression. Among the RGP-specific TFs were four Sox (*Sox1*, *Sox2*, *Sox9*, and *Sox21*), two Spalt (*Sall1* and *Sall3*), and two Tcf/Lef (*Tcf7l1* and *Tcf7l2*) family TFs, all linked to NSC maintenance and/or repression of neuronal differentiation. *Pax6*, previously characterized as a possible marker of RGPs (Götz et al., 1998), did not qualify as RGP-selective in the present analysis because microarray probes were discrepant. Indeed, Pax6 is expressed in many Tbr2+ IPs, especially aIPs, as well as RGPs (Englund et al., 2005). Other TFs, such as *Sox9*, are more specific RGP markers (Kaplan et al., 2017). Interestingly, among the RGP-selective RBPs, *Ngdn* (neuroguidin) regulates mRNA translation spatially and in response to signaling activity (Jung et al., 2006). Presumably, *Ngdn* may play a part in regulating local RGP translation, linked to rapid RNA transport (Pilaz et al., 2016). Among molecules for RNA degradation, *Pnrc2*, an adaptor in nonsense-mediated decay, was also RGP-selective.

#### RGP-Selective Signaling

Pathways were inferred from gene expression, with the caveat that protein signaling activity may not reflect mRNA expression. With this caveat in mind, pathway data (Figure 3B) showed that large numbers of RGP-selective genes were linked to cell cycle activity (*n* = 159) or DNA damage response (*n* = 10), the latter also active during the mitotic cycle (Petsalaki and Zachos, 2020). Genes that control NSC self-renewal (*n* = 29) or IP/neuronal differentiation (*n* = 18) were also numerous among RGP-selective genes. Other RGP-selective transcripts indicated functions such as membrane trafficking (*n* = 8), phagocytosis (*n* = 3), and GPCR trafficking (*n* = 1; *Cnih4*).

Several important intercellular and intracellular signaling pathways had one or more key components with RGP-selective expression. For example, multiple molecules in the NF-κB signaling pathway (Mitchell et al., 2016) showed RGP-selective expression (Figure 3C). The NF-κB transcriptional effectors p65 (*Rela*), p50 (*Nfkb1*), and TBL1 (*Tbl1x*) were all RGP-selective, as were several upstream positive and negative regulators. These findings indicate that the p65/p50-dependent NF-κB pathway is largely RGP-selective in developing neocortex. The function of NF-κB signaling in RGPs is primarily to maintain NSC identity and block IP genesis (Methot et al., 2013; Yamanishi et al., 2015). While the essential upstream activators of NF-κB signaling in developing cortex are unknown, this pathway can be engaged by TNF-R1 (*Tnfrsf1a*) activation or DNA damage (Fu et al., 2018; Van Quickelberghe et al., 2018). Activation of TNF-R1 (for example, by TNF-α, potentially released from activated microglia) may lead to NF-κB signaling or necroptosis (Khoury et al., 2020), the latter a type of apoptosis mediated by RIPK (*Ripk1*) (Supplementary Figure 3).

Additional pathways with RGP-selective components included Shh, Wnt, Ras-MAPK, hippo, IGF, FGF, and purinergic signaling (Supplementary Figure 3). The Shh signaling pathway functions in RGPs primarily to promote symmetric proliferative divisions (Dave et al., 2011), but must be modulated to prevent acquisition of ventral forebrain properties (Yabut et al., 2020). RGP-selective components of Shh signaling included *Gas1*, a Shh co-receptor that binds Shh and potentiates its activity; *Gli3*, a downstream effector of Shh that promotes proliferation and represses IP genesis; and *Tulp3*, an intracellular repressor of Shh signaling. The coordinated RGP-selective expression of both activators and inhibitors of signaling pathways was a theme observed with not only NF-κB and Shh, but also apoptosis, canonical Wnt, and hippo pathways (Supplementary Figure 3). In addition, RGPs were selectively enriched in components of the ephrin-B1 (*Efnb1*) signaling pathway, which represses neuronal differentiation (Qiu et al., 2008; Wu et al., 2009). *Efnb1*, its signaling partner *Rgs3*, and proposed target gene *Gpsm2* (LGN/Pins), were all RGP-selective. Moreover, we observed that *Ephb6*, a ligand for ephrin-B1, is highly expressed by IPs and PNs. Potentially, *Ephb6* from IPs and PNs might provide feedback to repress neuronal differentiation of RGPs. Since EphB6 lacks an intracellular kinase domain, its feedback to *Efnb1* could be an example of pure reverse signaling.

#### RGPs and Junctional Complexes

Near the apical (ventricular) surface, RGPs have robust adherens junctions (AJs) that form a belt-like zonula adherens (Taverna et al., 2014). But unlike most classic epithelial cells, RGPs do not form tight junction (TJ) barriers (Aaku-Saraste et al., 1996; Taverna et al., 2014; Veeraval et al., 2020). However, some TJ related proteins (TJRPs) are expressed in RGPs, such as ZO-1 (*Tjp1*) (Aaku-Saraste et al., 1996). IPs and neurons link to surrounding cells with AJ patches that are smaller and molecularly distinct from RGP AJs (Wilsch-Bräuninger et al., 2012). In RGPs, the zonula adherens is linked to a contractile ring of F-actin and non-muscle myosin type II (NM-II). RGPs and migrating neurons have gap junctions, which may be important for PN radial migration (Elias et al., 2007), but no gap junction genes were RGP-selective in our analysis.

#### Unique Molecular Composition of RGP AJs

**FIGURE 4:**
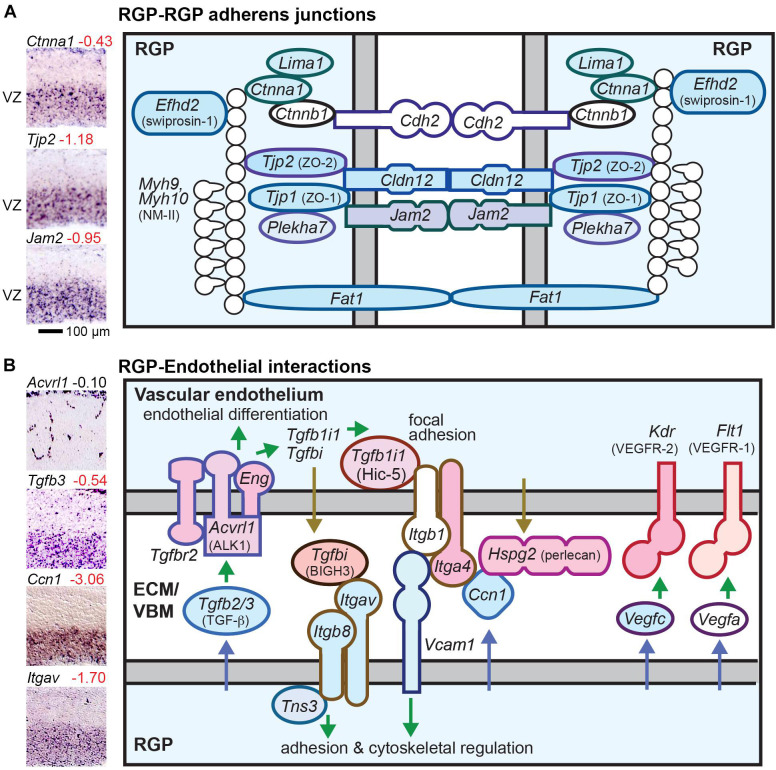
RGP adherens junctions and vascular interactions. **(A)** The RGP adherens junction includes many tight junction related proteins (TJRPs) encoded by genes such as *Tjp1*, *Tjp2*, and *Jam2*. The junctions form a continuous band around the RGP apical region, called the zonula adherens, which is promoted by Lima1. The adherens junction is linked to the actin cytoskeleton with a contractile ring of F-actin and NMII. *Cldn12* promotes paracellular calcium diffusion, and *Fat1* is a giant cadherin. **(B)** RGP interactions with endothelial cells. Key pathways for cortical vascular development include TGFβ, Integrin (α_v_β_8_), and VEGF produced by RGPs. Colored shapes indicate cell-type-specific genes; white fill indicates widely expressed genes.

We found that a large number of AJ and TJRP molecules are selectively expressed in RGPs (Figure 4A). Interestingly, RGPs expressed both “epithelial” (*Ctnna1*; αE-catenin) and “neural” (*Cdh2*; N-cadherin) AJ molecules (Figure 4A and Supplementary Table 2 column I). RGP-selective TJRPs included *Tjp1*, *Tjp2*, *Jam2*, and *Cldn12* (Figure 4A). The latter is an atypical claudin that promotes paracellular diffusion of calcium ions (Plain et al., 2020). Thus, although RGPs lack functional TJs and do not express *Ocln* (occludin), they express several TJRPs for enhanced adhesion and paracellular calcium diffusion. In addition, RGPs selectively expressed *Fat1*, which associates with AJs and promotes F-actin; *Rhoa*, a key regulator of AJ integrity; *Efhd2*, which stabilizes F-actin; *Lima1* (eplin), which promotes formation of the zonula adherens (Taguchi et al., 2011); and *Plekha7*, a zonula adherens-specific AJ adaptor that is repressed by *Insm1* to initiate IP delamination (Tavano et al., 2018). In addition, *Adgrv1*, an adhesion GPCR, is also selectively expressed by RGPs, but whether this molecule localizes in AJs is unknown. In sum, at least nine RGP-selective AJ molecules (shaded in Figure 4A) are down-regulated in the transition from RGP to aIP.

Apicobasal properties of RGPs such as the location of AJs are regulated in part by the apical polarity complexes PAR, CRB, and Mals/Pals. The PAR complex is composed of Pard3, Par6, atypical protein kinase C (aPKC), and Cdc42 (Kohjima et al., 2002). Some components of PAR complexes, such as *Prkci* (aPKCλ) and *Cdc42*, are essential to maintain AJs and apical surface integrity of the neocortex (Cappello et al., 2006; Imai et al., 2006). Perhaps surprisingly, then, none of the PAR complex molecules were RGP-selective. The CRB complex contains Crb, Pals1, and Patj proteins. Among these, only *Crb2* was RGP-selective. Indeed, previous studies have shown that *Crb2* is essential to maintain AJs and prevent premature RGP to IP differentiation (Dudok et al., 2016). No Mals/Pals molecules were RGP-selective.

The plasma membrane at the ventricular surface of RGPs, known as the apical plasma membrane, gives rise to the RGP primary cilium, and contains specific proteins for functions such as endocytic uptake and membrane remodeling. Among these, *Lrp2* (megalin) was RGP-selective, but *Prom1* was not (Our data suggest that *Prom1* is a marker of NSPCs, not NSCs). Among many dozens of known primary cilium molecules, only one (*Ift74*) was RGP-selective.

#### The Basal Surface and RGP Interactions With Leptomeninges

At the basal surface, RGPs attach to the basement membrane produced mainly by leptomeningeal cells (Supplementary Figure 4A) (Radner et al., 2012). Leptomeningeal-selective basement membrane genes included many isoforms of laminins (such as *Lama2*, *Lamb1*, and *Lamb2*) and collagens (such as *Col3a1*). RGPs selectively produced only a few ECM molecules, including *Vit*, *Ccdc80*, and *Bcan*. To attach to the basement membrane, RGPs require three basal attachment complexes built around dystroglycan (*Dag1*), which binds *Lama2*; GPR56 (*Adgrg1*), which binds *Col3a1*; and integrin-α_6_β_1_ (*Itga6*, *Itgb1*), which binds laminins and promotes focal adhesions. Of these, only *Dag1* showed RGP-selective expression, while *Adgrg1* and the integrin genes were also expressed in other cell types (Supplementary Figure 4A). Mutations in these basal attachment genes (such as *Dag1*) or their signaling pathways cause cobblestone-like cortical malformations (Myshrall et al., 2012; Radner et al., 2012). Meningeal cells also send an essential signal by producing *Bmp7*, which is necessary to maintain RGP attachment to the basement membrane (Segklia et al., 2012). Our analysis of ISH and microarray data indicates that RGPs express BMP7 receptor subunits ALK3 (*Bmpr1a*) and *Bmpr2*, although not RGP-specifically (Saxena et al., 2018) (Supplementary Figure 4A).

The basal plasma membrane of RGPs, which covers 99% of the RGP surface, expresses a basal polarity complex consisting of DLG, LGL, and SCRIB. This complex, together with endocytic adaptors *Numb* and *Numbl*, contribute to regulating the location of AJs; however, none of these molecules were expressed selectively in RGPs.

#### Notch Signaling

Activation of Notch receptors is essential for RGP self-renewal, and is driven by presentation of Delta-like 1 (*Dll1*), mostly from aIPs (Kawaguchi et al., 2008; Yoon et al., 2008; Nelson et al., 2013). Notch signaling occurs predominantly near the apical surface, and during mitosis is organized by the PAR complex. We found that *Notch1*, *Notch2*, and *Notch3* were selectively expressed by RGPs, along with *Hes5*, a downstream target gene that is activated by Notch signaling (Supplementary Figure 4B). On the IP side, we observed that *Dll1*, *Mib1* (an essential activator of *Dll1*), *Mfng* (a glycosyltransferase that modifies Dll1 and Notch), as well as *Hes6* and *Hey1* TFs, were IP-selective. Dll3 was bIP-selective, and acts cell autonomously to dampen Notch signaling. The Delta-Notch signaling interaction between IPs and RGPs is illustrated in Supplementary Figure 4B.

#### Mitochondria and Metabolism

Several mitochondrial molecules, including *Mrpl13*, *Mrpl35*, *Mrpl39*, *Ndufaf8*, *Timm44*, and others, were RGP-selective. Mitochondria are important in cortical development and their distribution within RGPs is regulated (Rash et al., 2018). Also, many molecules important in intermediary metabolism were RGP-selective (*n* = 44).

#### RGP Interactions With Endothelial Cells

Radial glial progenitors are known to play essential roles in cerebral cortex vascular development (Figure 4B) (Tata and Ruhrberg, 2018). For example, production of integrin-α_v_β_8_ (*Itgav* and *Itgb8*) by RGPs is essential for cortical vasculature development. Also, TGF-β and VEGF signals from RGPs promote vascular development. Many molecules that mediate RGP-endothelial interactions were selectively expressed by RGPs or endothelial cells, illustrating the extensive, highly specific interactions between these cell types (Figure 4B). Gene sets for vascular cell types are listed in Supplementary Table 9.

### Intermediate Progenitor Identity

IP-selective molecules were defined by significant enrichment in GFP+ cells on microarray, and predominant expression in the VZ/SVZ on ISH. Previous studies have reported that aIPs and bIPs have partially overlapping gene expression (Kawaguchi et al., 2008). To capture the distinct transcriptome profiles of aIPs and bIPs, we assessed IP-selective gene as “IP-a” if they were enriched predominantly in the VZ; “IP-ab” if in VZ and SVZ; or “IP-b” if mainly in the SVZ (Figure 1C). Accordingly, the aIP transcriptome consists of the union of IP-a and IP-ab genes, while the bIP transcriptome consists of IP-b plus IP-ab genes.

#### IP-Specific Transcripts

**FIGURE 5:**
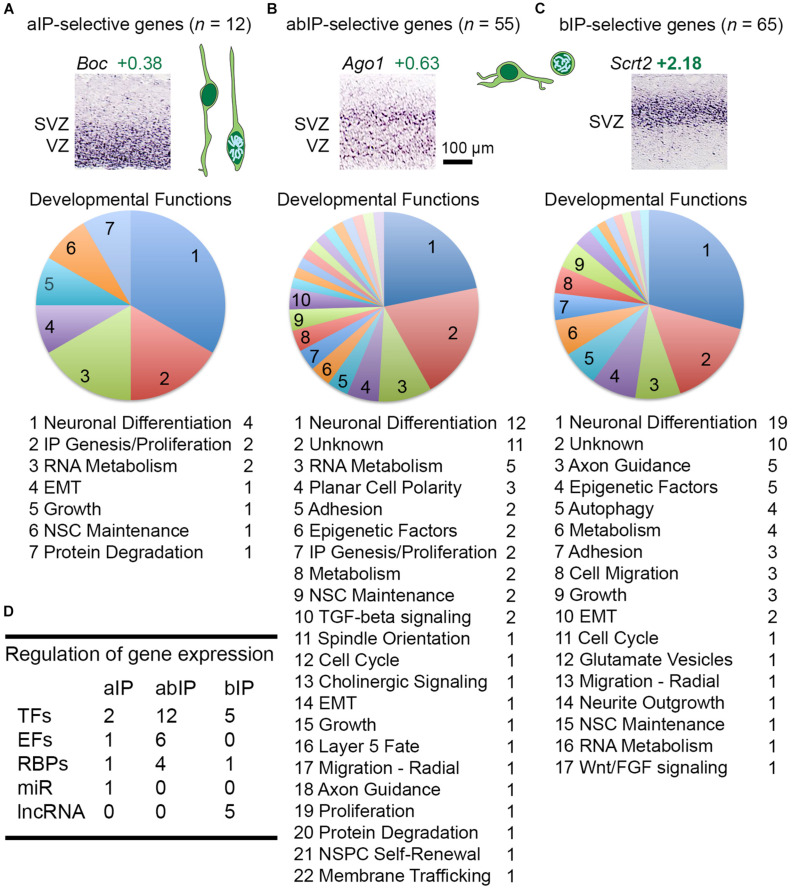
**(A–C)** IP-selective genes included those expressed mainly in VZ (IP-a genes), in VZ and SVZ (IP-ab), and in SVZ (IP-b), with examples for each. Neuronal development functions were highly represented in each category. **(D)** Table showing the number of gene regulatory molecules in each IP type.

Among 136 total IP-selective molecules, 12 were IP-a, 55 were IP-ab, and 69 were IP-b (Figures 5A–C and Supplementary Table 2). The largest functional category in each group was neuronal differentiation, consistent with previous evidence that Tbr2+ IPs are committed neurogenic progenitors that produce glutamatergic PNs (Hevner et al., 2006; Hevner, 2019). For gene regulation, each group of IP-selective genes included multiple TFs, such as *Neurog2* among IP-a genes, *Tbr2* in IP-ab genes, and *Neurod1* in IP-b genes (Figure 5D). These TFs illustrate that transcripts were ascertained for selective, but not absolutely specific expression. *Neurog2*, for example, fit criteria for IP-a enrichment, but is also expressed to some extent in RGPs (Kawaguchi et al., 2008). In contrast, *Tbr2* appears to be completely IP-specific, while *Neurod1* is expressed in bIPs and, to some extent, postmitotic neurons (Hevner et al., 2006). Other interesting regulators of gene expression selectively expressed in IPs included a microRNA (miR) host gene, *Mir17hg*, in aIPs; *Ago1*, an RBP that mediates mRNA silencing, in abIPs (Figure 5A); and multiple lncRNAs in bIPs. The epigenetic factors selectively expressed by IPs have been described (Elsen et al., 2018).

#### TF Regulation in IPs

The transitions between RGPs, IPs, and PNs appear to be highly discrete. In accordance with this view, regulatory mechanisms that rapidly control the expression of key TFs in IPs have been identified. While *Pax6* is crucial to IP genesis and *Tbr2* expression (Quinn et al., 2007), *Pax6* is downregulated and deactivated in IPs, in part by feedback repression from *Tbr2* (Elsen et al., 2018), and in part due to dephosphorylation by protein phosphatase-1 (Yan et al., 2007). Similarly, *Neurog2*, another driver of IP genesis and *Tbr2* expression (Ochiai et al., 2009), is rapidly downregulated in IPs by *Cbfa2t2* (MTGR1), an IP-specific transcriptional coregulator that is first induced by *Neurog2*, then binds and inactivates *Neurog2* (Aaker et al., 2009). Indeed, decreased *Neurog2* activity is essential for further differentiation from IPs to PNs (Aaker et al., 2009). These examples illustrate the principle that differentiation in the RGP-IP-PN lineage requires rapid, active up- and downregulation of critical genes.

#### Adhesion and Apoptosis Pathways in IPs

Compared to RGPs, IPs markedly down-regulated proliferation, AJ components, and RGP-specific pathways such as NF-κB, hippo, and necroptosis. Not only were AJ components overall downregulated in IPs, but the isoform of α-catenin also changed from *Ctnna1* (αE-catenin) in RGPs, to *Ctnna2* (αN-catenin) in IPs and differentiating PNs. Conversely, aIPs and bIPs selectively expressed *Ptprk*, a homophilic adhesion molecule. These data revealed a rapid, profound change in both the strength and quality of AJs occurs concomitantly with RGP-IP differentiation. Interestingly, while necroptosis pathways were reduced in IPs, apoptosis of IPs may occur by other pathways, mediated by “dependence receptors” that promote apoptosis if ligand is not bound (see below). Consistent with this idea, IPs and neurons express *Ppp2r2b*, which drives apoptosis in response to growth factor deficiency.

#### Shh and Wnt Signaling Pathways in IPs

Shh co-receptor *Boc* was selectively expressed in aIPs (Figure 5A), rather than *Gas1* as in RGPs (Supplementary Figure 3B). The canonical Wnt signaling pathway (involving regulation of β-catenin signaling) was likewise modified in IPs, for example, by selective upregulation of *Fzd1* in the PN lineage. Previous studies have shown that in multipolar bIPs, canonical Wnt signaling is transiently downregulated (Boitard et al., 2015). Our molecular analysis suggested that this change might be mediated by *Bcl6* (Bonnefont et al., 2019), expressed selectively in the IP-PN lineage; and by *Shisa2*, a bIP-selective, cell-autonomous inhibitor of Wnt and FGF signaling (Furushima et al., 2007). However, non-canonical Wnt signaling, especially the Wnt-PCP pathway, appears massively upregulated in IPs (see below).

#### Delta-Notch Signaling in IPs

Notch pathway molecules were differentially and selectively expressed between not only RGPs and IPs, but also between aIPs and bIPs. Expression of *Dll1* was aIP-selective (Supplementary Figure 4B), while *Dll3* was bIP-selective. Like *Dll1*, *Dll3* is a Delta-like ligand that is fucosylated by *Mfng*, but *Dll3* functions cell-autonomously to block Notch activation and thus consolidate neuronal differentiation of bIP cells (Serth et al., 2015). At the same time, *Bcl6* (which is selectively expressed in the IP-PN lineage, beginning in aIPs) functions to repress Notch signaling and, together with *Bcor* (an abIP-selective gene), represses *Hes5* (Tiberi et al., 2012). *Hey1* (abIP-selective) represses *Hes1* and *Gas1*, while promoting self-renewal of NSPCs (Heisig et al., 2012; Weber et al., 2014). Thus, *Hey1*, together with *Boc* (replacing *Gas1*) in the Shh pathway, may support IP division despite overall reduced proliferative activity of IPs compared to RGPs.

#### Extracellular Matrix and Vascular Interactions

Intermediate progenitors do not interact with the meningeal basement membrane, but do associate with blood vessels, especially in the SVZ (Javaherian and Kriegstein, 2009; Stubbs et al., 2009). Interestingly, we observed that bIPs selectively express *Mfap4*, an RGD-containing ECM component that may be a ligand for integrins and collagen. We found that *Adgrg1* (GPR56), although best known for mediating RGP interactions with the meninges (Supplementary Figure 4A), is enriched in abIPs. We speculate that *Adgrg1* and/or *Mfap4* may mediate the association of IPs with blood vessels. Also, abIPs selectively expressed *Ltbp3* and *Mfap2* (MAGP-1), both of which stabilize TGF-β, and thus potentially enhance TGF-β signaling from RGPs to blood vessels (Figure 4B).

#### IP Cell Migration

Previous studies have shown that as IPs differentiate from aIP to bIP subtype, they migrate from the VZ where they have “short radial” (Gal et al., 2006) or “pin-like” (Ochiai et al., 2009) morphology, to the SVZ where bIPs remodel to multipolar morphology. The aIPs initially maintain contact with RGPs at apical AJs (Taverna et al., 2014), which are lost in the transition to bIP. After final mitosis, new PNs exhibit multipolar morphology, select an axonal process, convert to bipolar morphology, then migrate into the cortical plate. During the multipolar phase, bIPs and new PNs undergo “multipolar migration” characterized by frequent extension and retraction of short processes, and short-range slow tangential migration in the SVZ and IZ (Tabata and Nakajima, 2003).

#### Epithelial-Mesenchymal Transition

One proposed mechanism for neural precursor migration from the VZ is epithelial-mesenchymal transition (EMT) (Itoh et al., 2013). Consistent with this idea, we observed that several critical genes for EMT (Nieto et al., 2016; Aznar et al., 2018) are selectively expressed in IPs, including *Srsf2* in aIPs, *Akna* in abIPs, and *Scrt2* and *Ccdc88c* (Daple) in bIPs (Supplementary Table 2). By ISH, scattered *Scrt2*-expressing cells were observed in the VZ, likely indicating cells in transition from aIP to bIP (Figure 5C). In addition, *Serpini1* (neuroserpin), which is expressed in the IP-PN lineage beginning in the SVZ, may contribute to EMT (Matsuda et al., 2016). Partial or specialized forms of EMT are common in biology (Nieto et al., 2016). In addition, ATP and Ca(2+) signaling may also regulate migration from VZ to SVZ (Liu et al., 2008).

#### Multipolar Migration

During the multipolar phase, we found that bIPs selectively expressed *Chn2* (β2-chimerin; Figure 1C), a Rac-GAP, as well as *Prex1* (P-Rex-1), a Rac-GEF. Both *Chn2* and *Prex1* have been linked to regulation of multipolar migration, indicating the importance of *Rac1* (which is widely expressed) in this process. Interestingly, *Chn2* links EphA receptor signaling with Rac1 inactivation to suppress migration (Takeuchi et al., 2009), while *Prex1* activates Rac1 and stimulates migration (Li et al., 2019). Since several EphA molecules (such as *Epha4*, *Epha5*) are expressed in the SVZ and IZ, our data suggest that EphA activation restricts migration. Lateral dispersion of PN clones away from source RGPs is also regulated by EphA signaling (Torii et al., 2009). Interestingly, lateral dispersion of neural precursors thus occurs before axogenesis and bipolar migration.

Other known regulators of bIP and new PN migration include *Rnd3* (Pacary et al., 2011), which we found was selectively expressed by bIPs; and *Rnd2*, which was expressed by new PNs (Heng et al., 2008). Another factor that may regulate IP migration is the peptide CCK. The receptor CCK-1R (*Cckar*), reported to mediate repulsive responses to CCK (Giacobini et al., 2004), was selectively expressed on bIPs. The *Cck* ligand was the expressed by PNs in the cortical plate (CP). Potentially, CCK signaling may restrict IPs from entering the CP.

#### Cholinergic Signaling

Intermediate progenitors express cholinergic receptor subunits, including *Chrna3* selectively in abIPs, and *Chrnb2* in IPs and new neurons beginning in the SVZ. Cholinergic receptors can be activated *in vivo* to provoke inward Ca(2+) currents (Atluri et al., 2001). However, the functional significance of cholinergic signaling remains unknown.

#### Wnt-PCP Pathway

Planar cell polarity is a conserved mechanism to polarize sheets of cells in the tangential plane, for example, to orient bristles on the fly body (Butler and Wallingford, 2017). The molecular components of PCP include “core” and “Fat–Dachsous–Four-jointed” (Ft–Ds–Fj) modules, which may interact concurrently or sequentially. Arising by asymmetric endocytosis and endosomal trafficking, PCP ultimately reorganizes the actin cytoskeleton to control cellular morphology. One key mechanism that can orient PCP is Wnt signaling gradients (Yang and Mlodzik, 2015).

**FIGURE 6:**
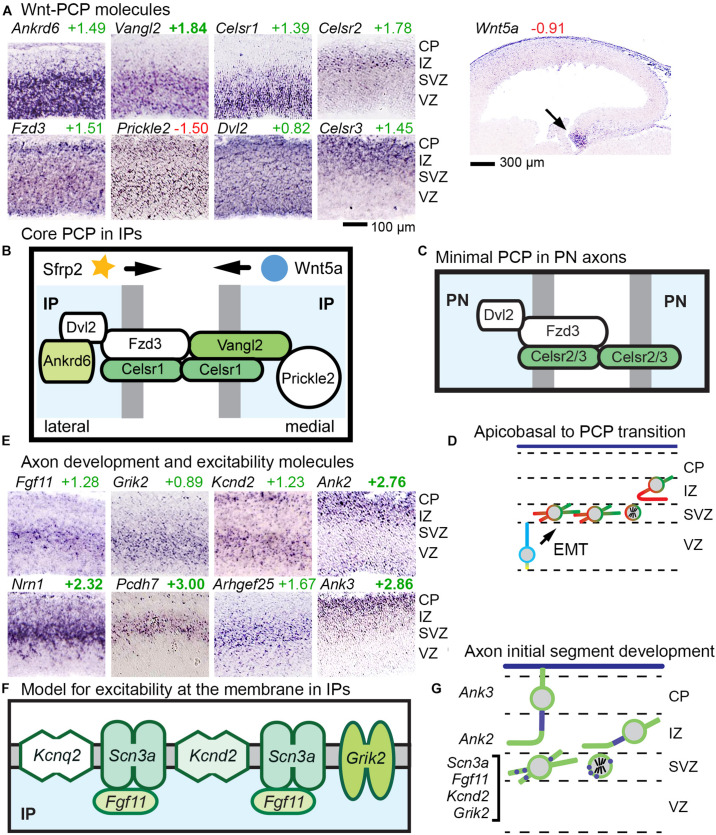
Wnt-PCP and axon differentiation pathways in IPs. **(A)** *Ankrd6*, *Vangl2*, and *Celsr1* showed IP-specific expression; *Fzd3*, *Prickle2*, and *Dvl2* were broadly expressed; *Celsr2* and *Celsr3* were expressed in postmitotic PNs. **(B)** Proposed model of Wnt-PCP signaling complexes in IPs, possibly patterned by gradients of Wnt signaling, such as *Wnt5a* from the hem. *Sfrp2* from the antihem might also shape Wnt gradients. Colored shapes indicate IP-selective genes, and white fill indicates widely expressed molecules. **(C)** Proposed model of “minimal PCP” in PNs. Unlike IPs, PNs do not produce a full complement of core PCP molecules, but do express *Fzd3*, *Celsr2/3*, and *Dvl2*. **(D)** Proposed model of transition from apicobasal polarity (blue–yellow) to PCP (red–green) in aIP to bIP differentiation. In this model, PCP is initially patterned in IPs, and transmitted to postmitotic neurons in the IZ. **(E)** Molecules for axon development and excitability were expressed in the SVZ (including *Fgf11*, *Grik2*, *Kcnd2*, *Nrn1*, *Pcdh7*, and *Arhgef25* as shown), while genes for axon initial segment stabilization were expressed in postmitotic PNs (*Ank2* and *Ank3*) of the IZ and CP. **(F)** Proposed model of axon-like excitability patch in IPs. **(G)** Proposed model for the development of excitability beginning in IPs. Patches of excitable membrane may be produced in IPs, and rapidly assembled into axon initial segments in new postmitotic PNs.

Core PCP is implemented by six molecules (*Fzd*, *Vangl*, *Celsr*, *Dvl*, *Prickle*, and *Ankrd6*), some of which have multiple gene isoforms. We found that a suite of three core PCP molecules were expressed selectively in abIPs: *Celsr1*, *Vangl2*, and *Ankrd6*. Together with broadly expressed isoforms of other core PCP components — including *Fzd3*, *Prickle2*, and *Dvl2* — IPs thus uniquely acquire a full complement of core PCP components (Figures 6A,B). Notably, *Fzd3* is the isoform most frequently implicated in core PCP, although other *Fzd* isoforms can be involved as well.

While several mechanisms may contribute to orienting the directionality of PCP, one is by response to Wnt gradients, known as Wnt-PCP signaling (Yang and Mlodzik, 2015). In developing cortex, Wnt signaling gradients (Machon et al., 2007) are established by caudomedial expression of multiple Wnts and *Rspo2* (which potentiates Wnts) in the cortical hem and hippocampus; and by *Sfrp2* (a Wnt antagonist) from the rostrolateral antihem (Supplementary Figure 1). The Wnt most associated with PCP signaling in mammals is *Wnt5a*, expressed in the hem (Figure 6A). Other Wnts and Fzds show diverse expression patterns that demonstrate the complexity of Wnt signaling in developing cortex (Supplementary Figure 5).

Our findings suggest that concurrent with EMT, IPs undergo a profound change in polarity, from apicobasal to planar (Figure 6D). In PNs, no *Ankrd6* or *Vangl* isoforms were detected, and instead of *Celsr1* as in IPs, *Celsr2* and *Celsr3* were expressed in PNs (Figure 6A). These molecular data suggest that PNs may express a minimal “maintenance” form of PCP, initially oriented in IPs, to promote axon fasciculation (Figure 6C). Importantly, mutations in core PCP molecules have been associated with defects of axon growth and connectivity in the forebrain (Hakanen et al., 2019).

#### Axogenesis and Excitability

Axon selection by cortical PNs is thought to occur shortly after they are generated from IPs, concurrently with the transition from multipolar to bipolar morphology (Namba et al., 2014). Interestingly, we observed that multiple genes associated with axogenesis, neurite growth, and excitability were expressed by bIPs (Figure 6E).

Among the bIP-selective genes for axogenesis were *Pcdh7*, which initiates axon outgrowth in retina; *Arhgef25*, which drives axon formation and growth; *Nrn1* (neuritin-1), an activity-induced cell surface protein that promotes axon growth; *Sstr2*, which stimulates axon outgrowth upon activation by somatostatin (Le Verche et al., 2009); *Bcar1*, an adaptor protein linked to neurite outgrowth; *Igfs8*, a cell surface protein associated with neurite outgrowth; *Ppp2r3c*, a regulatory component of protein phosphatase 2A (PP2A), which is linked to axogenesis; and *Dusp14*, a phosphatase that inhibits MAPK signaling and negatively regulates axon growth (references in Supplementary Table 2, column Z). Another critical regulator of axon formation in neocortex, *Rapgef1*, was expressed in the IP-PN lineage beginning in bIPs. The selective expression of not only activators, but also an inhibitor of axon growth suggests that axogenesis is a regulated process that begins in bIPs.

#### Intrinsic Excitability

A recent study unexpectedly found that voltage-gated sodium channel *SCN3A* (Na_V_1.3) is expressed by Tbr2+ IPs in developing human cortex (Smith et al., 2018). In the present study, *Scn3a* could not be evaluated because *Scn3a* ISH data are not available for embryonic mouse. Nevertheless, we did find other excitability molecules expressed selectively in bIPs (Figure 6E). These included *Grik2* (GluK2/GluR6), which causes membrane depolarization upon glutamate binding; *Kcnd2* (K_V_4.2), which mediates repolarization; and *Fgf11*, a member of the FGF-homologous factor family, whose members function as intracellular modulators of voltage-gated sodium channels, and may localize with them to the AIS (Goldfarb et al., 2007; Pablo and Pitt, 2016). In addition, *Kcnq2* (K_V_7.2) was also expressed in IPs, albeit not selectively. Molecular markers of stabilized axon initial segments, such as *Ank2*, *Ank3*, and *Cacna2d1*, were not expressed until PNs reach the IZ and CP (Figure 6E). Thus, despite the fact that IPs lack axons or initial segments, IPs may function to promote excitability even before the axon is formed (Figures 6F,G). We speculate that together, IP-expressed ion channels and *Fgf11* may form excitable membrane patches (Figure 6F), with properties similar to the *Ank3*-independent, immature AIS which accumulates voltage-gated sodium channels and K_V_7.2 (Sánchez-Ponce et al., 2012; Yamada and Kuba, 2016). Also, *Nfasc* (neurofascin), another molecule linked to axon initial segment stabilization, was expressed in bIPs; but this function of *Nfasc* is thought to be *Ank3*-dependent.

#### Glutamatergic Signaling in IPs

**FIGURE 7:**
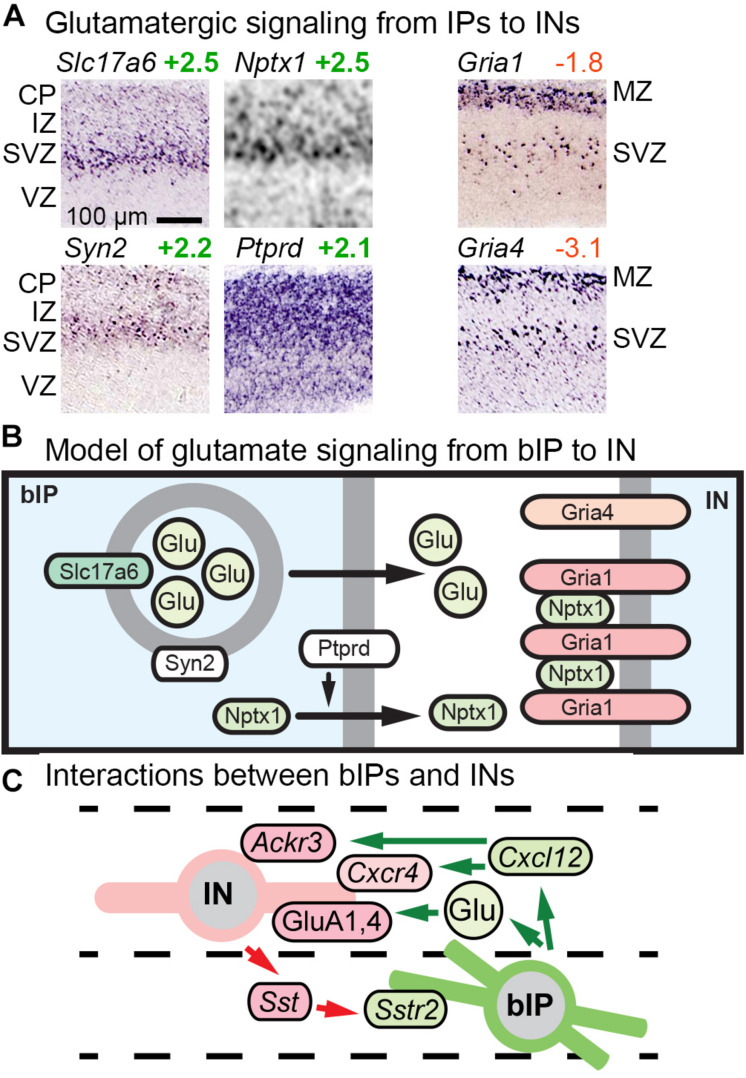
Glutamate signaling and other bIP-IN interactions. **(A)** bIPs in the SVZ express molecules for glutamatergic signaling including *Slc17a6* (VGLUT2), *Nptx1*, *Syn2*, and *Ptprd*; while migrating INs express AMPA receptors (*Gria1*, *Gria4*). **(B)** Proposed model for glutamatergic signaling from bIPs to INs. Interestingly, bIPs produce *Nptx1*, a secreted protein that clusters AMPA receptors. **(C)** Glutamate signaling is one of several interactions between IPs and INs.

Previous studies reported that bIPs express *Slc17a6* (VGLUT2), a vesicular glutamate transporter that packages glutamate for release as a neurotransmitter (Kawaguchi et al., 2008). Our analysis confirmed that bIPs selectively express *Slc17a6*, and further revealed that bIPs also express *Syn2* (synapsin II), a synaptic vesicle molecule enriched in the IP-PN lineage beginning in bIPs (Figure 7A). Thus, IPs may be prepared to release glutamate vesicles. In addition, we found that bIPs selectively express *Nptx1*, a secreted clustering factor for GluA1-type glutamate receptors (Figure 7A). Moreover, bIPs express *Ptprd* (RPTPδ), a tyrosine phosphatase that functions to promote release of *Nptx1*. Interestingly, GluA1 (*Gria1*), as well as GluA4 (*Gria4*), are selectively expressed by migrating INs (Figure 7A). Thus, bIPs have some properties of glutamate-releasing neurons, along with the capacity to promote glutamate receptor clustering on adjacent cells, in this case INs (Figure 7B). Overall, glutamate signaling may be another mechanism of interaction between IPs and INs, along with *Cxcl12* and *Sstr2* (Figure 7C).

#### Axon Guidance Molecules in IPs

**FIGURE 8:**
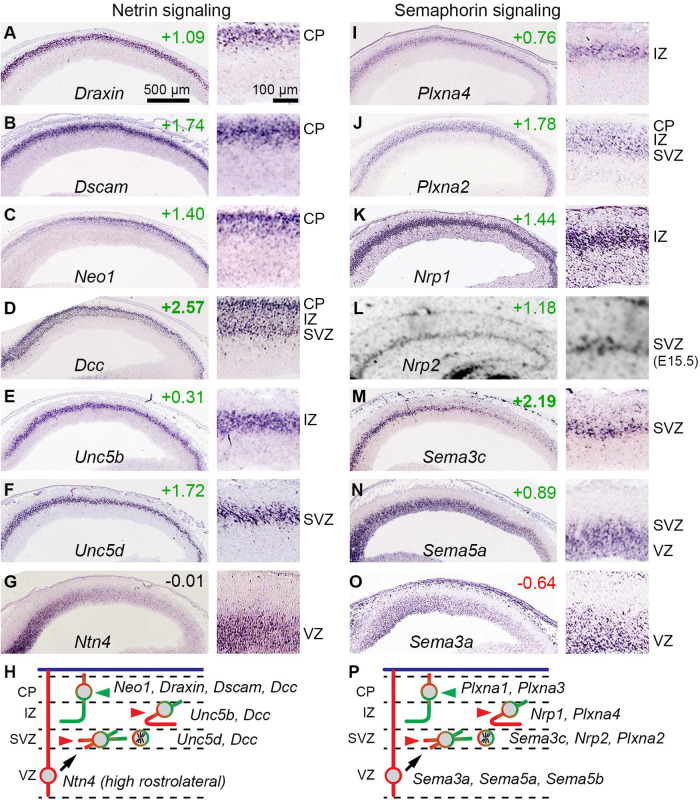
Netrin and semaphorin signaling involving IPs. **(A–G)** Expression of selected netrin signaling molecules, including bIP-selective *Unc5d* **(F)**. Each panel shows low **(left)** and high **(right)** magnification views of cortex; rostral is to the left. **(H)** Proposed model for netrin signaling involving *Ntn4* from RGPs in a high rostral gradient, and responsive PNs in each histologic zone. **(I–O)** Expression of selected semaphorin signaling molecules, including bIP-selective genes *Plxna2* **(J)**, *Nrp2* **(L)**, and *Sema3c* **(M)**; and abIP-selective *Sema5a* **(N)**. **(P)** Proposed model for semaphorin signaling among cell types.

Previous studies also reported that IPs express axon guidance receptors, including *Unc5d* (same gene as *Svet1*) and *Plxna2* (Kawaguchi et al., 2008). Our analysis confirmed and expanded this repertoire, finding that IPs also selectively express *Sema3c*, *Sema5a*, and *Nrp2*. These molecules belong to netrin and semaphorin guidance pathways (Figure 8).

#### Netrin Signaling and IPs

Netrins are secreted factors that attract or repel axons, depending on the receptor: For *Ntn1*, the most studied netrin, *Dcc* and *Neo1* are attractive, while *Unc5* family molecules interact with *Dcc* to form repulsive receptors (Boyer and Gupton, 2018; Yamagishi et al., 2021). Other molecules that interact with the netrin system include *Dscam*, a netrin co-receptor with *Dcc*; and *Draxin*, a secreted factor that binds *Dcc* to modulate thalamocortical axon guidance. In addition, *Flrt2* and *Flrt3* secreted ligands can bind *Unc5d* and *Unc5b* receptors, respectively, to mediate repulsive cues for cell migration (Yamagishi et al., 2021).

Among netrins, *Ntn1* and *Ntn4* have similar receptor binding and interactions (Qin et al., 2007). *Ntn1* is not expressed in embryonic neocortex, but *Ntn4* expression has been reported in E14.5 VZ (Yin et al., 2000; Ayoub et al., 2011). We confirmed expression of *Ntn4* in the VZ on ISH, and furthermore observed a high rostrocaudal to low caudomedial gradient (Figure 8G). (*Ntn4* was not detected at significant levels on microarray, presumably for technical reasons). These findings suggested that *Ntn4* may be important in cortical development. Indeed, *Ntn4* deficient rats show reduced thalamocortical innervation (Hayano et al., 2014).

*Dcc* was highly enriched in the IP-PN lineage (log_2_FC =+2.57), and was expressed in the SVZ, IZ, and CP, consistent with bIPs and PNs (Figure 8D). Since bIPs also express *Unc5d* (Figure 8F), they presumably respond to *Ntn4* as a repulsive guidance cue. This repulsive response is likely maintained for new PNs in the IZ, which express *Unc5b* (Figure 8E). In contrast, PNs in the CP did not express *Unc5* molecules, except for very low levels of *Unc5a* in the most superficial CP cells (not shown). PNs in the CP did, however, express high levels of *Dcc*, *Neo1* (in a high caudal gradient), *Dscam*, and *Draxin* (Figures 8A–D), suggesting that axons from PNs already in the CP on E14.5 (early-born PNs) respond to *Ntn4* as an attractive cue (Figure 8H). These findings suggest that netrin signaling may play an important early role in bIP polarization and axon guidance.

#### Semaphorin Signaling and IPs

In this repulsive signaling system, semaphorins are ligands, neuropilins are ligand-binding receptors, and plexins are signal-transducing co-receptors (reviewed by Alto and Terman, 2017; Toledano et al., 2019). Previous studies have shown that this system is important in the development of callosal axons, which are repelled toward the midline by graded expression of rostrolateral factors, including *Sema3a*, a secreted semaphorin. We confirmed that *Sema3a* is expressed in a high rostrolateral gradient in the VZ, and further found that *Sema3a* is selectively expressed by RGPs (Figure 8O). The *Sema3a* receptor, *Nrp1* (Hossain et al., 2019), and the co-receptor *Plxna4*, were both expressed by new PNs in the IZ (Figures 8I,K). Thus, many new PNs in the IZ on E14.5 express *Nrp1*/*Plxna4* repulsive receptors for *Sema3a*, consistent with callosal axon phenotype (Figure 8P).

Many additional semaphorins and receptors were expressed by specific cell types. Interestingly, like *Sema3a* in RGPs, other semaphorins were expressed in high rostrolateral gradients, including *Sema5a* in abIPs (Figure 8N) and *Sema3c* in bIPs (Figure 8M). Also, *Sema5b* was expressed specifically by RGPs, but no gradient was apparent (not shown). The receptor for *Sema5a* consists of *Nrp2* with *Plxna2*, both expressed selectively by bIPs (Figures 8J,L); signaling through this receptor may be further potentiated by *Ranbp9*, a bIP-selective scaffold protein that augments plexin-A activity. *Sema3c* has multiple potential receptors. *Sema5b*, a transmembrane semaphorin, functions as a repulsive cue for *Nrp1*+/*Plxna1*+ corticofugal axons to prevent their entry into the VZ. While the complexity of signaling using multiple semaphorins (each with multiple receptors) defies simple predictions about effects on axon guidance, it seems clear at least that the high rostrocaudal gradients of secreted semaphorins (*Sema3a*, *Sema3c*, and *Sema5a*) may have additive or redundant effects, especially on cortical axons destined to cross the midline (Suárez et al., 2014; Ku and Torii, 2020).

Molecules in the Robo-Slit system are also extremely important in neocortical axon guidance, but were not selectively expressed in IPs or RGPs, only in postmitotic neurons.

#### Apoptosis-Inducing Dependence Receptors

Previous studies have reported high levels of apoptosis in the SVZ (Blaschke et al., 1996; Thomaidou et al., 1997), recently attributed to asymmetric apoptosis of IP daughter cells (Mihalas and Hevner, 2018). In contrast to RGPs, which were specifically enriched in necroptosis pathways (Supplementary Figure 3A), IPs showed no selective enrichment for apoptosis effectors. However, we noted that IPs and new PNs in the IZ express multiple dependence receptors, defined as transmembrane receptors that trigger cell death if not bound by ligand (Negulescu and Mehlen, 2018). Dependence receptors in bIPs included *Unc5d*, *Dcc*, *Ntrk3*, and *Epha4*. As noted above, *Unc5d* and *Dcc* are netrin receptors. *Ntrk3*, which is highly (but not selectively) expressed in bIPs in the SVZ, is the receptor for *Ntf3*, and is part of a neurogenic feedback mechanism from PNs to progenitor cells that promotes a switch to upper layer fates (Parthasarathy et al., 2014). *Epha4* is a significant driver of cortical progenitor cell proliferation (North et al., 2009). These findings suggest that PN numbers are regulated by competitive mechanisms that utilize dependence receptors.

#### Gene Sets for Other Cell Types

Gene sets for all neural and non-neural cell types in E14.5 mouse neocortex are presented in Supplementary Tables 3–10. The criteria for assignment of each cell type, and the specific Supplementary Table, are given in the “Materials and Methods” section.

## Discussion

In the present study, we produced cell-type-specific gene sets for E14.5 mouse neocortex, and analyzed the transcriptomes of RGPs and IPs in detail. We found that IPs express suites of genes for processes such as EMT, PCP, neuron polarization, axogenesis, excitability, and glutamate signaling. Since IPs are still dividing and do not have axons, our findings raise new questions about how processes such as neuron polarization and axogenesis could begin in progenitor cells. In addition, it is important to recognize the limitations of our approach to assigning IP-selective genes, and question whether the combination of transcriptomes and ISH assigns genes to specific cell types accurately. Also, it is important to keep in mind that mRNA expression does not always correlate with protein abundance and post-translational regulation.

Compared to previous studies, our approach identified relatively large numbers of cell-type-selective genes, especially for IPs (Kawaguchi et al., 2008; Cameron et al., 2012; Telley et al., 2016). This outcome likely reflected the robust quantities of RNA used for our bulk analysis. One pitfall was the interpretation of ISH images to distinguish between bIPs in the SVZ, and new PNs in the IZ: the SVZ and IZ overlap histologically, and some genes are expressed in both cell types. In particular, genes involved in neuron polarization, axon guidance, and excitability, might seem more likely to be neuron-selective, rather than IP-selective. On the other hand, other studies have independently substantiated IP expression of some axon guidance and excitability genes. Axon guidance molecules *Plxna2* and *Unc5d* were reported in IPs by single-cell analysis (Kawaguchi et al., 2008). Also, core PCP molecule *Celsr1* was confirmed as an IP gene (Telley et al., 2016). And most significantly, *SCN3A* was previously colocalized with Tbr2 in IPs of developing human neocortex (Smith et al., 2018). Overall, the coherence of our results and independent confirmations increase confidence in the conclusions.

### Epithelial-Mesenchymal Transition (EMT)

Epithelial-mesenchymal transition is a flexible process, usually involving some or most of the same key EMT molecules, such as *Scrt2* (Nieto et al., 2016). In a previous study, EMT of new neocortical neurons was attributed to the activity of *Scrt1* and *Scrt2*, and was proposed to occur by transcriptional repression of *Cdh1* (E-cadherin) (Itoh et al., 2013). In the present study, we found that EMT begins at the step when aIPs delaminate from the ventricular surface to become bIPs (Figure 6D). Also, we did not detect *Cdh1* expression in E14.5 mouse neocortex by microarray or ISH, making *Cdh1* an unlikely target of EMT-related transcriptional repression. Furthermore, we identified *Scrt2* selectively in bIPs, with some expression in aIPs (Figure 5C), consistent with EMT occurring mainly in IPs, not RGPs. These findings imply that bIPs have some mesenchymal-like properties.

### Core PCP in IPs and Minimal PCP in PNs

Our results revealed coordinate abIP-selective expression of *Celsr1*, *Ankrd6*, and *Vangl2*, completing, along with more broadly expressed molecules, a core PCP program in IPs (Figures 6A,B). In contrast, postmitotic PNs apparently expressed only a rudimentary set of core PCP molecules, including *Fzd3* (the *Fzd* most often linked to PCP) along with *Celsr2* or *Celsr3* instead of *Celsr1* (Figure 6C). The PCP polarization of IPs is consistent with previous studies showing that, although PCP was originally described in epithelia, mesenchymal-like cells can also have PCP, for example, in convergent extension during gastrulation and neurulation, and in limb bud morphogenesis (Yang and Mlodzik, 2015). Our findings raise the following questions: (1) What is the function of core PCP in IPs? (2) What mechanisms orient PCP in IPs? (3) Can PCP that is established in IPs be transmitted to daughter PNs?

The functions of core PCP in developing neocortex have previously been linked mainly to directional growth of axons (reviewed by Hakanen et al., 2019). The most severe axon phenotypes are seen in mice lacking *Fzd3* (Wang et al., 2002; Hua et al., 2014), or *Celsr2* and *Celsr3* (Qu et al., 2014). Striking similarities between the *Fzd3* and *Celsr2/Celsr3* knockout phenotypes suggest an interaction between these molecules, consistent with our model for “minimal” PCP in PN axons (Figure 6C). On this basis, we propose that *Celsr* molecules are plausible candidates for mediating “touch and go” interactions between new PN axons and established tracts (Namba et al., 2014). The original “touch and go” model (Namba et al., 2014) proposed that TAG1 (*Cntn2*) and *Lyn* mediate axon interactions; however, *Cntn2* null mice have no significant axonal defects, and in our assays, *Lyn* was detected only in non-neural cells of E14.5 neocortex.

Of the IP-enriched PCP genes, mice lacking *Celsr1* have microcephaly, due to reduced numbers of IPs and PNs (Boucherie et al., 2018). This phenotype was previously attributed to RGP defects, but as *Celsr1* expression is IP-selective, defects in IPs would seem more likely. For example, it could be that PCP is required in IPs for axon orientation, and axon failure causes apoptosis. Deficiency of *Vangl2* in mice (*Lp*/*Lp*) caused severe cortical thinning (Lake and Sokol, 2009), but cortex-specific *Vangl2* knockout caused a much different phenotype, with partial agenesis of the corpus callosum and hippocampal commissure (Dos-Santos Carvalho et al., 2020). Mice lacking *Ankrd6* have no reported defects of cortical development, although subtle changes of PCP were reported in the inner ear (Jones et al., 2014). Together, the data suggest that in IPs, core PCP is important to maintain IP numbers, and to facilitate growth of axon pathways. These phenotypes may be related because, if core PCP serves to polarize IPs, then it is possible that “a failure to correctly polarize the budding axon leads to abortive axonal outgrowth” (Wang et al., 2002) and cell death.

We propose that PCP polarization of IPs serves to pre-orient and thereby optimize polarization of PNs, improving the efficiency of axon selection and growth. Previously, PCP has been shown to regulate progenitor cell activities in flies, fish, and mammals. In flies and zebrafish, PCP can orient cell divisions, which is interesting because IPs divide with mostly horizontal cleavage planes, while RGPs divide with vertical cleavage planes (Smart, 1973; Englund et al., 2005; Kowalczyk et al., 2009). In mammals, progenitor cells in developing epidermis express PCP components that are internalized and redistributed during mitosis, in a process that is important for patterning of the skin and hair follicles (Devenport et al., 2011).

### Wnt-PCP Signaling in IPs

Wnt-PCP is a signaling pathway conserved from flies to mammals, in which Wnt gradients orient tissue polarity by binding to *Fzd* receptors that are part of PCP complexes (Yang and Mlodzik, 2015; Humphries and Mlodzik, 2018). In developing neocortex, high caudomedial gradients of canonical Wnt signaling have been documented (Machon et al., 2007), and *Wnt5a* (a non-canonical Wnt frequently associated with PCP) is, like several other Wnts (Supplementary Figures 1, 5), expressed mainly in the cortical hem, a caudomedial patterning center (Figure 6A). Also, *Rspo1* and *Rspo2*, which bind Wnts to enhance signaling potency, are likewise expressed in the hem. Conversely, *Sfrp2*, a secreted molecule that binds Wnts to inhibit their signaling, is highly expressed in the antihem (rostrolateral patterning center) (Supplementary Figure 1A) and may sharpen Wnt gradients (Humphries and Mlodzik, 2018). Moreover, “transient downregulation of canonical Wnt/β-catenin signaling during the multipolar stage” may facilitate a switch to non-canonical, Wnt-PCP signaling in bIPs (Boitard et al., 2015).

Overall, the data suggest that an intracortical gradient of *Wnt5a* and other Wnts could potentially orient PCP in IPs by Wnt-PCP signaling. But could this system orient axons? Previous studies have demonstrated that Wnt-PCP signaling can specify axon orientation and steer axon growth in diverse species (Onishi et al., 2014). For example, Wnt-PCP polarizes and guides axons in *Caenorhabditis elegans* (Ackley, 2014) and zebrafish (Sun et al., 2016). Together with the genetic evidence implicating PCP in cortical axon development, these observations suggest that Wnt-PCP signaling in IPs may orient the future growth of PN axons, possibly in conjunction with axon guidance molecules.

### IPs and Netrin Signaling

We (Figure 8F) and Tarabykin et al. (2001) and Kawaguchi et al. (2008) found that bIPs selectively express *Unc5d* (*Svet1*), a netrin receptor transducing repulsive responses. One proposed function of *Unc5d* is to regulate cell migration through interactions with *Flrt2*; however, *Unc5d* null mice exhibit no histological defects in cortical layer formation (Yamagishi et al., 2011). Moreover, *Ntn4* produced by RGPs in a high rostrocaudal gradient (Figure 8G; see also Yin et al., 2000) would be expected to bind *Unc5d* and repel IP processes. Interestingly, *Ntn4* deficient rats show aberrant thalamocortical innervation (Hayano et al., 2014), but it remains unclear how the guidance of PN axons is affected.

### Axon Polarity Can Be Regulated Prior to Mitosis

Previous studies have shown that axon polarity can be oriented in neural progenitor cells prior to mitosis and axon outgrowth. In *C. elegans*, multiple guidance molecules function to regulate polarity coordinates that are inherited by daughter neurons (Killeen and Sybingco, 2008; Adler et al., 2014). In chick neural crest, polarity generated prior to mitosis can be inherited by dorsal root ganglia neurons through a mechanism involving *Septin7*, which labels sites for re-initiation of process growth following mitosis (Boubakar et al., 2017). In neocortex, *Septin7* is essential for callosal and corticospinal axon growth (Ageta-Ishihara et al., 2013). Together, these observations support the possibility that PN axon polarization is initiated in IPs prior to final mitosis.

### Excitability in IPs

Surprisingly, several genes linked to neuronal excitability are expressed in IPs (Figures 6E–G). This finding intersects with a recent report that *SCN3A*, a voltage-gated sodium channel, is expressed in IPs and is required for cortical morphogenesis (Smith et al., 2018). What role might IP excitability play in cortical development? One possibility is that IPs express excitability genes to enhance interactions with migrating INs (Figure 7). A second possibility is that IPs accumulate excitability molecules in order to “prime” PNs for rapid development of excitability after mitosis. Third, excitability may enhance survival and differentiation of IPs and new PNs. For example, it has been shown that depolarization recruits *Dcc* to the plasma membrane and enhances axon growth (Bouchard et al., 2008).

### Polarization of IPs as a Mechanism to Enhance PN Axon Development

Recent observations suggest that new PNs become polarized and accelerate axon growth upon contacting a pre-existing PN axon, prompting the “touch and go” model (Namba et al., 2014). In our modification of this model (Figures 6B,C), we propose that *Celsr* genes make contact at the axon surface, while *Fzd3* transduces the signal as a form of Wnt-PCP signaling for axon fasciculation. In addition, *Ntn4-Unc5d* might also orient the axon prior to IP mitosis. In sum, we propose that IPs are oriented by Wnt-PCP and netrin signaling, start to become excitable upon reaching the SVZ as bIPs, and produce “pre-polarized” PNs that interact with adjacent existing axons to rapidly integrate into cortical circuitry.

## Conclusion

We have ascertained gene sets for cell types in E14.5 mouse cortex, and found that IPs selectively express genes involved in core PCP, axogenesis, axon guidance, excitability, and glutamate signaling. On this basis, we propose new neurodevelopmental functions for IPs, in optimizing axon development and integration into cortical pathways. These novel functions add to previously known IP roles in amplifying neurogenesis, shaping regional and laminar identity of PNs, and signaling to INs and RGPs.

## Materials and Methods

### Animals

No new mice were used for this study; only data from previous microarrays and ISH were analyzed. The *Tbr2*-GFP mice for cell sorting and microarrays were described previously (Bedogni et al., 2010; Nelson et al., 2013; Elsen et al., 2018).

### Identification of Differentially Expressed Genes (DEGs) in Microarray Experiments

Genes from the microarray experiment (Supplementary Table 1) were selected as DEGs if they showed significantly different expression (unadjusted *p* < 0.05) between sorted cell populations (GFP+ and GFP−), and were expressed above a minimal detection threshold (2.279), determined empirically to accord with ISH detection. For genes with multiple probes, the gene was excluded if different probes conflicted by indicating significant enrichment in both GFP+ and GFP− cells. If multiple probes were differentially expressed and in agreement, the probe with highest absolute value of log_2_FC was used to represent the gene for integrated analysis (Supplementary Table 2).

### Selection of Cell-Type-Selective Genes by Microarray and ISH Criteria

For each cell type, specific criteria of expression on microarray and by ISH were utilized. The ISH data were from public open databases, or previous studies. The public databases were Genepaint (Visel et al., 2004), Allen Brain Atlas Developing Mouse Brain^1^, and BGEM (Magdaleno et al., 2006). If no ISH data were available, the zonal expression data of Ayoub et al. (2011) were used.

### Cajal-Retzius (C-R) Neurons

C-R neuron-selective genes (Supplementary Table 3) were significantly enriched in GFP+ cells, and were expressed exclusively or predominantly in the marginal zone on ISH. Established markers of C-R neurons (*Trp73*, *Calb2*, and *Reln*) were confirmed as C-R markers. Additional genes were also screened from candidate C-R neuron markers (Yamazaki et al., 2004), of which two (*Cacna2d2* and *Rcan2*) were included.

### Choroid Plexus

Genes (Supplementary Table 8) were selected on the basis of enrichment in *Tbr2*-GFP− cells on microarray, and predominant expression in choroid plexus epithelium (not fibrovascular core) on ISH. *Ttr*, a known marker of choroid plexus, met these criteria and was included.

### Interneurons (INs) and Subtypes

Interneurons (Supplementary Table 8) were enriched in *Tbr2*-GFP− cells on microarray, and showed expression predominantly in the marginal zone and SVZ. Although most IN subtypes do not differentiate until postnatal ages, we also assessed peptide markers of IN subtypes (*Calb1*, *Npy*, *Pvalb*, *Sst*, and *Tac1*). All except *Pvalb* (which was below the detection threshold) were significantly expressed and enriched in Tbr2-GFP− cells. Putative IN genes from previous studies were also screened (Batista-Brito et al., 2008; Marsh et al., 2008).

### Intermediate Progenitors

IP genes (Supplementary Table 3) were assessed as listed below for each subtype. Putative IP genes from previous studies were also screened (Kawaguchi et al., 2008; Cameron et al., 2012; Telley et al., 2016) along with novel targets.

### Apical Intermediate Progenitor Enriched Genes (IP-a)

These genes (Supplementary Table 3) were selected on the basis of significant enrichment in Tbr2-GFP+ cells on microarray, and expression predominantly in the VZ on ISH.

### Apical and Basal Intermediate Progenitor Enriched Genes (IP-ab)

Genes for this set (Supplementary Table 3) were selected on the basis of significant enrichment in Tbr2-GFP+ cells on microarray, and largely balanced expression predominantly in the VZ and SVZ on ISH.

### Basal Intermediate Progenitor Enriched Genes (IP-b)

These (Supplementary Table 3) were selected for significant enrichment in Tbr2-GFP+ cells on microarray, and expression primarily in the SVZ on ISH.

### Microglia

Genes (Supplementary Table 10) were selected on the basis of enrichment in Tbr2-GFP− cells, expression in cells scattered in all cortical zones, and literature linking the gene to microglia. Widely used microglial markers *Aif1* (Iba-1 gene), *Cx3cr1*, and *Cd68* met these criteria and were among the included genes.

### Meninges (Leptomeninges)

Meningeal genes (Supplementary Table 9) were enriched in Tbr2-GFP− cells on microarray, and showed predominantly leptomeningeal expression on ISH. Putative meningeal markers were also screened (DeSisto et al., 2020).

### Neuronal Differentiation in the Cortical Plate (N-cp)

These genes (Supplementary Table 4), representing general neuronal differentiation in the CP, were selected by significant enrichment in Tbr2-GFP+ cells on microarray, and expression predominantly in the CP on ISH. They were also expressed in neuronal differentiation zones of other forebrain areas besides cortex.

### Neuronal Differentiation in the Intermediate Zone (N-iz)

These genes (Supplementary Table 4), representing general neuronal differentiation beginning in the IZ, were selected by significant enrichment in Tbr2-GFP+ cells on microarray, and expression predominantly in the IZ, or IZ and CP, on ISH. They were also expressed in neuronal differentiation zones in other forebrain areas besides cortex.

### Neuronal Differentiation in the Subventricular Zone (N-svz)

These genes (Supplementary Table 4) were highly enriched in Tbr2-GFP+ cells on microarray, and were expressed in the SVZ and IZ, or SVZ, IZ, and CP on ISH. They were also expressed in similar zones in other forebrain areas.

### Neuronal Differentiation in the Ventricular Zone (N-vz)

These markers of general neuronal differentiation (Supplementary Table 4) were enriched in Tbr2-GFP+ cells on microarray, and showed expression in the VZ, SVZ, IZ, and CP on ISH. They were also expressed in differentiating neurons in other forebrain areas.

### Oligodendroglial Progenitor Cells

Oligodendrocytes do not differentiate until postnatal ages. the possibilities that oligogenic lineages might exist in E14.5 cortex, or might differentiate prematurely in mutant mice, prompted us to select markers of oligodendroglia in postnatal cortex as candidate oligodendrocyte identity genes (Supplementary Table 8). Two genes (*Olig1* and *Pdgfra*) were significantly enriched in Tbr2-GFP− cells on microarray, and were expressed by scattered cells in progenitor and differentiation compartments (mainly rostrolateral), suggesting that a few immature oligodendrocyte precursors are present in E14.5 neocortex.

### Projection Neuron Differentiation in the Cortical Plate (PN-cp)

These genes (Supplementary Table 3), representing relatively specific differentiation of projection neurons in the CP, were selected by significant enrichment in Tbr2-GFP+ cells on microarray, and expression predominantly in the CP on ISH. They were not highly expressed in other areas of the telencephalon besides cortex, such as the striatum.

### Projection Neuron Differentiation in the Intermediate Zone (PN-iz)

These genes (Supplementary Table 3), representing specific differentiation of projection neurons beginning in the IZ, were selected by significant enrichment in Tbr2-GFP+ cells on microarray, and expression predominantly in the IZ, or IZ and CP, on ISH. They were not expressed in other areas of the telencephalon besides cortex, such as the striatum.

### Projection Neuron Differentiation in the Subventricular Zone (PN-svz)

These genes (Supplementary Table 3), representing specific differentiation of projection neurons beginning in the SVZ, were selected by significant enrichment in Tbr2-GFP+ cells on microarray, and expression predominantly in the SVZ and IZ, or SVZ, IZ, and CP, on ISH. They were not highly expressed in other areas of the telencephalon besides cortex.

### Projection Neuron Differentiation in the Ventricular Zone (PN-vz)

These genes (Supplementary Table 3), representing relatively specific differentiation of projection neurons beginning in the VZ, were selected by significant enrichment in Tbr2-GFP+ cells on microarray, with expression in the VZ, SVZ, IZ, and CP on ISH. These genes were not expressed at high levels in the VZ of subcortical areas of the telencephalon.

### Subplate

The goal was to identify glutamatergic pioneer neurons in the histological subplate. Accordingly, genes (Supplementary Table 3) were selected by significant enrichment in *Tbr2*-GFP+ cells on microarray, and predominant expression in the subplate on ISH. Proposed markers of the embryonic subplate (Hoerder-Suabedissen et al., 2013) were also screened.

### Vascular Cells

These genes (Supplementary Table 9) were enriched in Tbr2-GFP− cells on microarray, and were expressed by cells lining vascular spaces. They were further annotated by reference to literature for each gene, and divided into endothelial and pericyte groups.

### Vascular Endothelial Cells

These genes (Supplementary Table 9) were significantly enriched in Tbr2-GFP− cells on microarray, were expressed in elongated cells of the vascular endothelium, and were confirmed as endothelial markers by literature references.

### Vascular Pericytes

These genes (Supplementary Table 9) were significantly enriched in Tbr2-GFP− cells on microarray, were expressed in scattered cells along blood vessels, and were confirmed as pericyte markers by literature references.

### Blood Cells

These genes (Supplementary Table 10) were enriched in Tbr2-GFP− cells on microarray, and were expressed by cells within vascular spaces. They were further annotated by reference to literature for each gene, and divided into erythrocyte, lymphocyte, and monocyte groups where possible from available literature.

### Neural Stem Cells

Candidate NSC markers were screened from previous studies (Easterday et al., 2003; Andreotti et al., 2019). NSC markers (Supplementary Table 7) met criteria for RGPs (enriched in GFP− cells on microarray, and localized predominantly in the VZ).

### Callosal Projection Neurons

Genes in CPN Signature clusters (Molyneaux et al., 2015) were screened. Genes (Supplementary Table 6) were selected if they demonstrated enrichment in Tbr2-GFP+ cells, regardless of ISH localization.

### Corticothalamic Neurons

Genes in CThN Signature clusters (Molyneaux et al., 2015) were selected for evaluation, and were included (Supplementary Table 6) if they demonstrated significant enrichment in *Tbr2*-GFP+ cells, and were localized mainly in the cortical plate. Genes with predominant expression in progenitor zones were excluded.

### Subcerebral Projection Neurons

Genes in SCPN Signature clusters (Molyneaux et al., 2015) were screened, were included (Supplementary Table 6) if they showed enrichment in Tbr2-GFP+ cells, and were expressed mainly in the IZ and CP. Notable excluded genes were *Sox5* and *Bcl11b*, which were classified as Mixed cell-type genes by Molyneaux et al. (2015).

### Upper Layers 2–4

Genes previously implicated in genesis of upper layers in publications were selected (Supplementary Table 6) if they showed significant expression and enrichment in *Tbr2*-GFP+ cells, and were expressed predominantly in progenitor zones (VZ and SVZ) and/or IZ, possibly extending into the upper CP. Notable exclusions were *Cux1* and *Cux2*, due to non-enrichment in *Tbr2*-GFP + cells (possibly reflecting expression in INs). *Tac2* (Figure 2D) was below the detection threshold on microarray, presumably for technical reasons, but was retained as an upper layer marker.

### Lower Layers 5–6

Lower layers are comprised of mainly corticothalamic and subcerebral projection neurons. Genes were selected (Supplementary Table 6) if they met criteria for either of those cell types.

### Hem and Antihem

Genes (Supplementary Table 5) were selected if they showed predominant expression in the patterning center by ISH. No microarray criteria were applied.

### Rostral and Caudal Identity

These gene sets (Supplementary Table 5) have been developed and expanded from previous studies (Bedogni et al., 2010; Elsen et al., 2013). Most recently, we have stratified rostral and caudal gene sets into CP, IZ, SVZ, and VZ subsets, to precisely evaluate rostrocaudal patterning of differentiating neurons and progenitors in different zones. The criteria for each subset are described next.

### Rostral Identity in the Cortical Plate (R-cp)

By ISH, these genes (Supplementary Table 5) were expressed predominantly in the CP and subplate, at higher levels in rostral than in caudal neocortex. On microarray, genes were enriched in GFP+ cells, or were not enriched in either cell group.

### Rostral Identity in the Intermediate Zone (R-iz)

By ISH, these genes (Supplementary Table 5) were expressed predominantly in the IZ, or IZ and CP, at higher levels in rostral than in caudal neocortex. On microarray, these genes were enriched in GFP+ cells, or were not enriched in either cell group.

### Rostral Identity in the SVZ (R-svz)

By ISH, these genes (Supplementary Table 5) were expressed predominantly in the SVZ, at higher levels in rostral than in caudal neocortex. On microarray, these genes were enriched in GFP+ cells, or were not enriched in either cell group.

### Rostral Identity in the VZ (R-vz)

By ISH, these genes (Supplementary Table 5) were expressed predominantly in the VZ, at higher levels in rostral than in caudal neocortex. No microarray criteria were used, because these genes could theoretically be expressed in RGPs, RGPs and aIPs, or aIPs.

### Caudal Identity

Caudal identity in each zone (Supplementary Table 5) was assessed using the same criteria as for rostral identity, except that the gene was expressed at higher levels in caudal than in rostral cortex.

### Proliferation Genes

Genes (Supplementary Table 7) were first evaluated from known proliferation markers across cell types (Whitfield et al., 2006). Additional proliferation markers were selected if they were functionally linked primarily to cell cycle, and matched the expression of validated markers (Whitfield et al., 2006) in mainly RGPs.

### Quiescence Genes

These (Supplementary Table 7) were aggregated from multiple previous studies.

#### RBPs

These were designated according to McKee et al. (2005), or more recent studies documenting RNA-binding activity, as cited for individual genes.

#### TFs

These were designated according to Gray et al. (2004), or more recent studies documenting TF function, as cited for individual genes.

## Data Availability Statement

Publicly available datasets were generated for this study. This data can be found here: NCBI GEO accession number GSE22371 (GPL1261) at www.ncbi.nlm.nih.gov/geo/query/acc.cgi?acc=GPL1261.

## Ethics Statement

The animal study was reviewed and approved by The University of Washington Institutional Animal Care and Use Committee (where the original animal experiments were done).

## Author Contributions

FB and RH: study conception and design, acquisition of data, analysis and interpretation of data, critical revision, and final approval of the version to be published. RH: drafting of manuscript. Both authors contributed to the article and approved the submitted version.

## Conflict of Interest

The authors declare that the research was conducted in the absence of any commercial or financial relationships that could be construed as a potential conflict of interest.

## Footnotes

1 http://developingmouse.brain-map.org/
