# Supplementary figures and images for: Cell-Type-Specific Gene Expression in Developing Mouse Neocortex: Intermediate Progenitors Implicated in Axon Development
Source: Front Mol Neurosci. 2021 Jul 12;14:686034. doi: 10.3389/fnmol.2021.686034 (PMC8313239; doi:10.3389/fnmol.2021.686034)

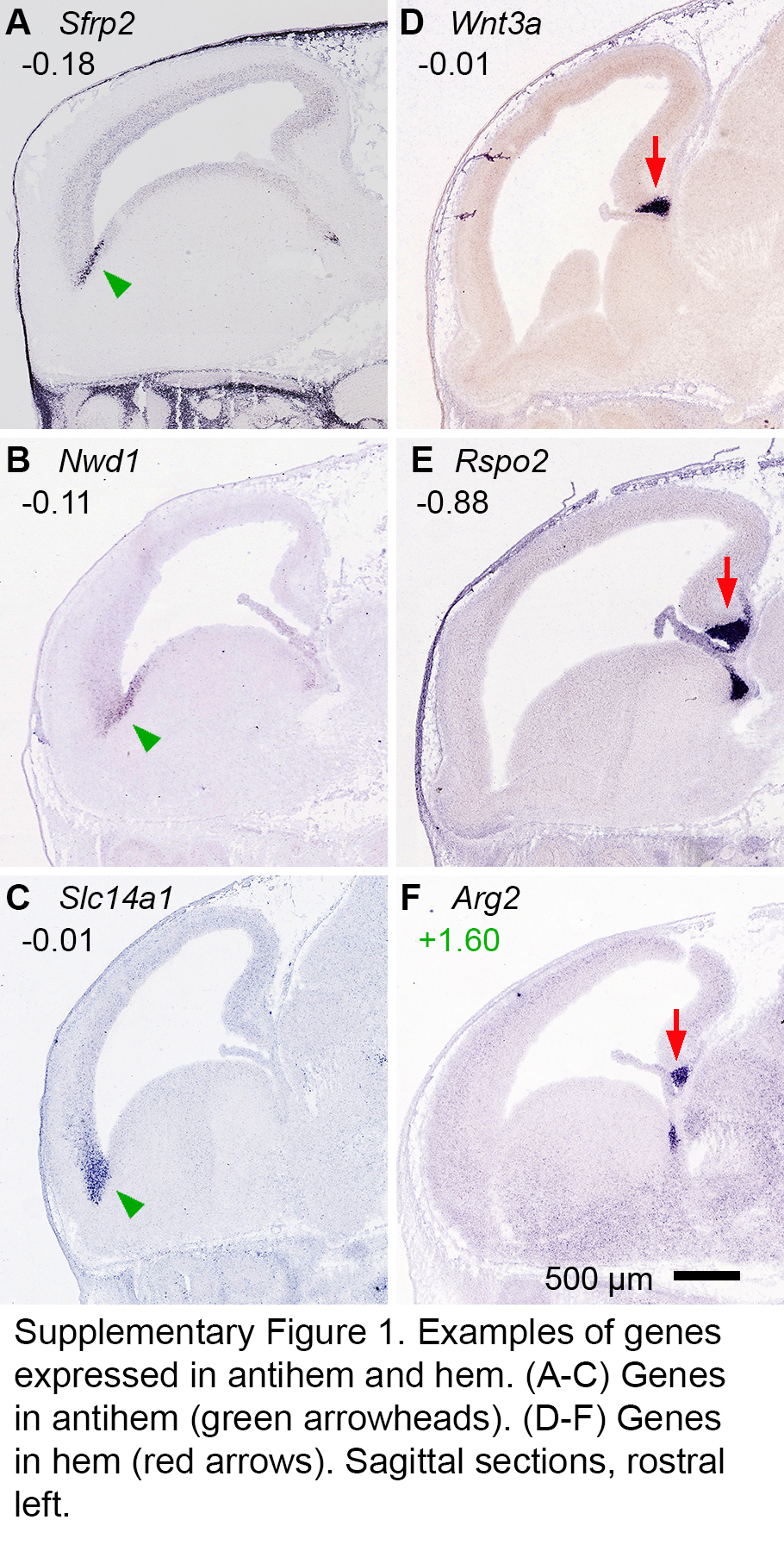

Supplement: Supplementary file 1 [file Image_1.JPEG]

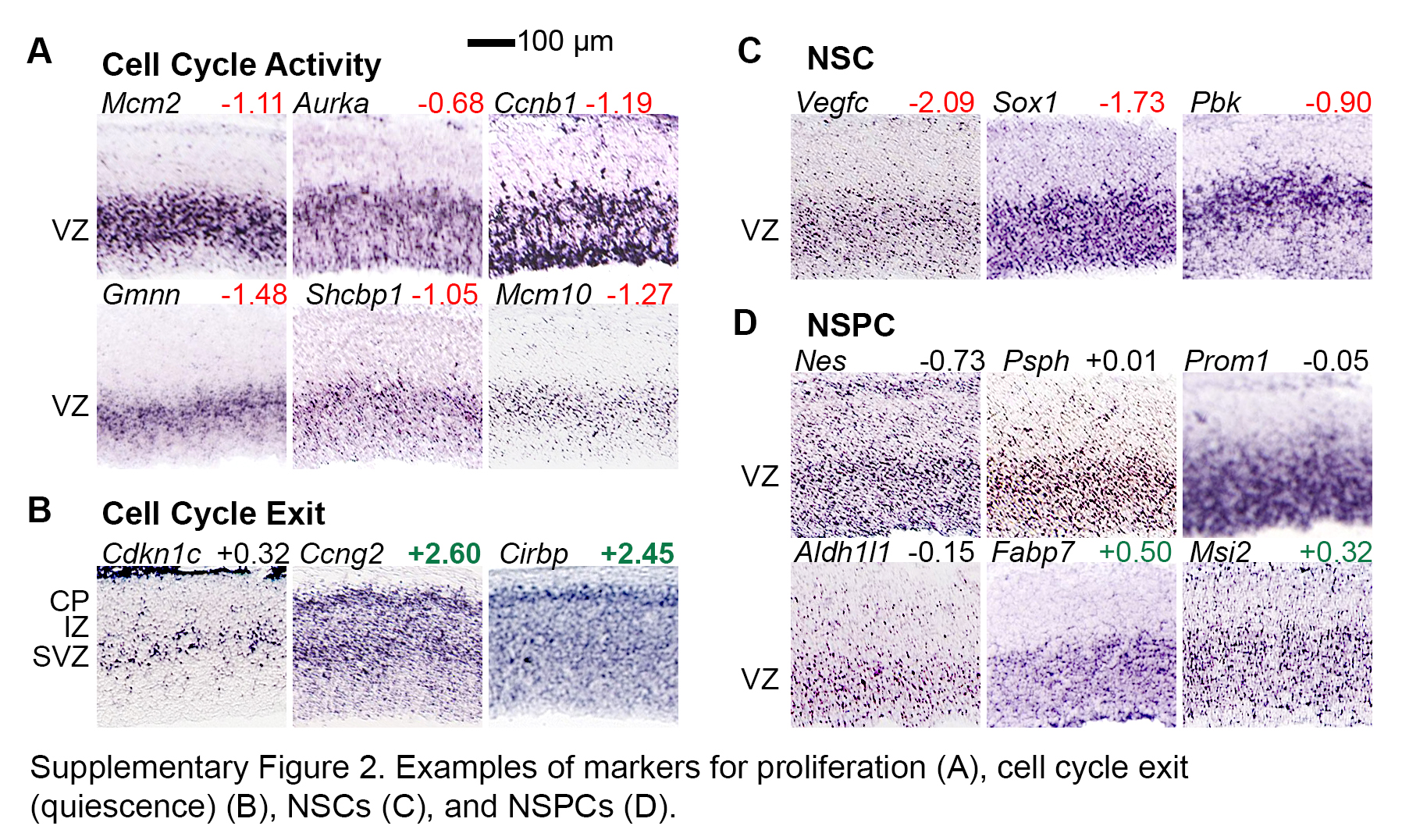

Supplement: Supplementary file 2 [file Image_2.JPEG]

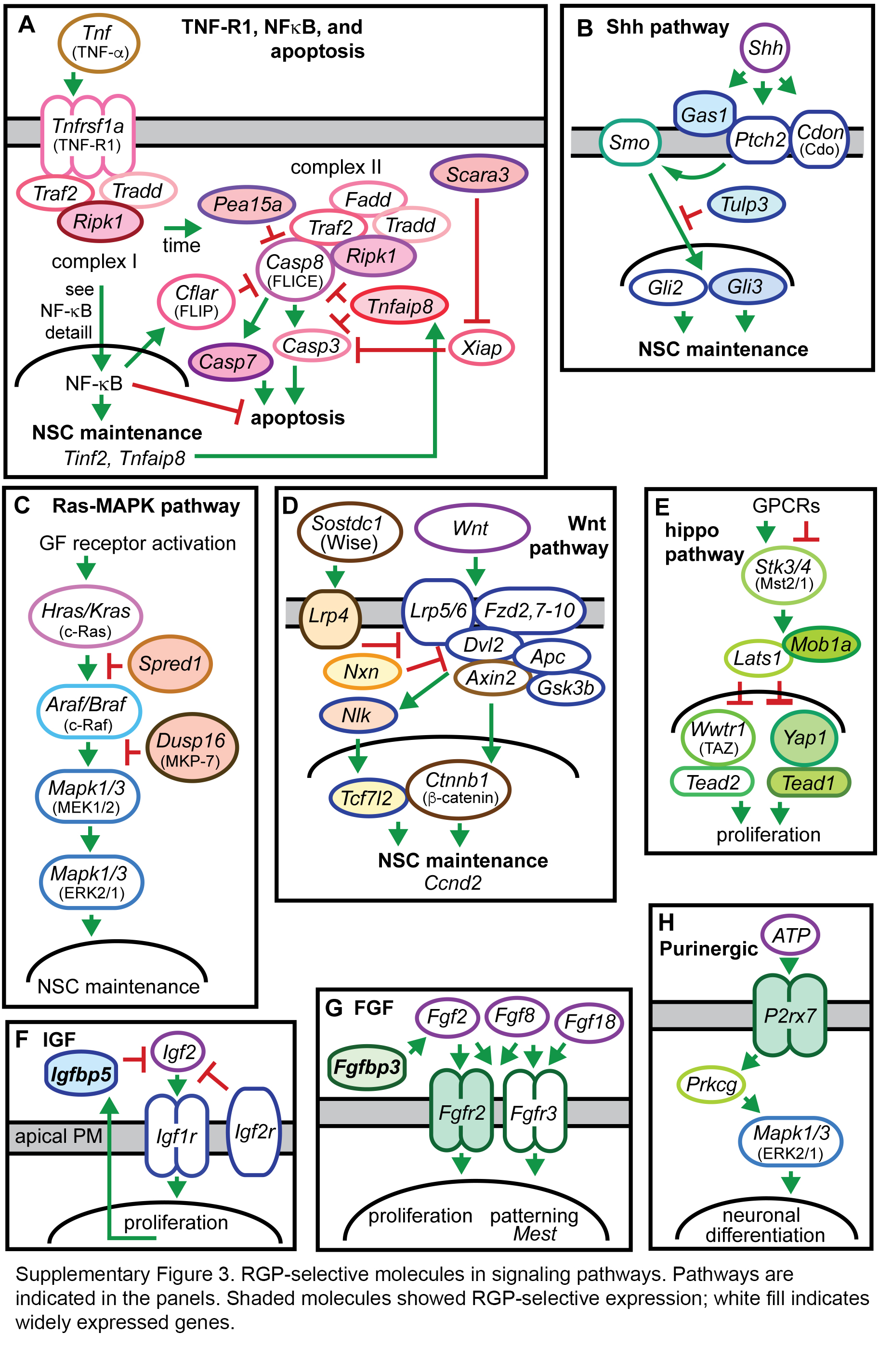

Supplement: Supplementary file 3 [file Image_3.JPEG]

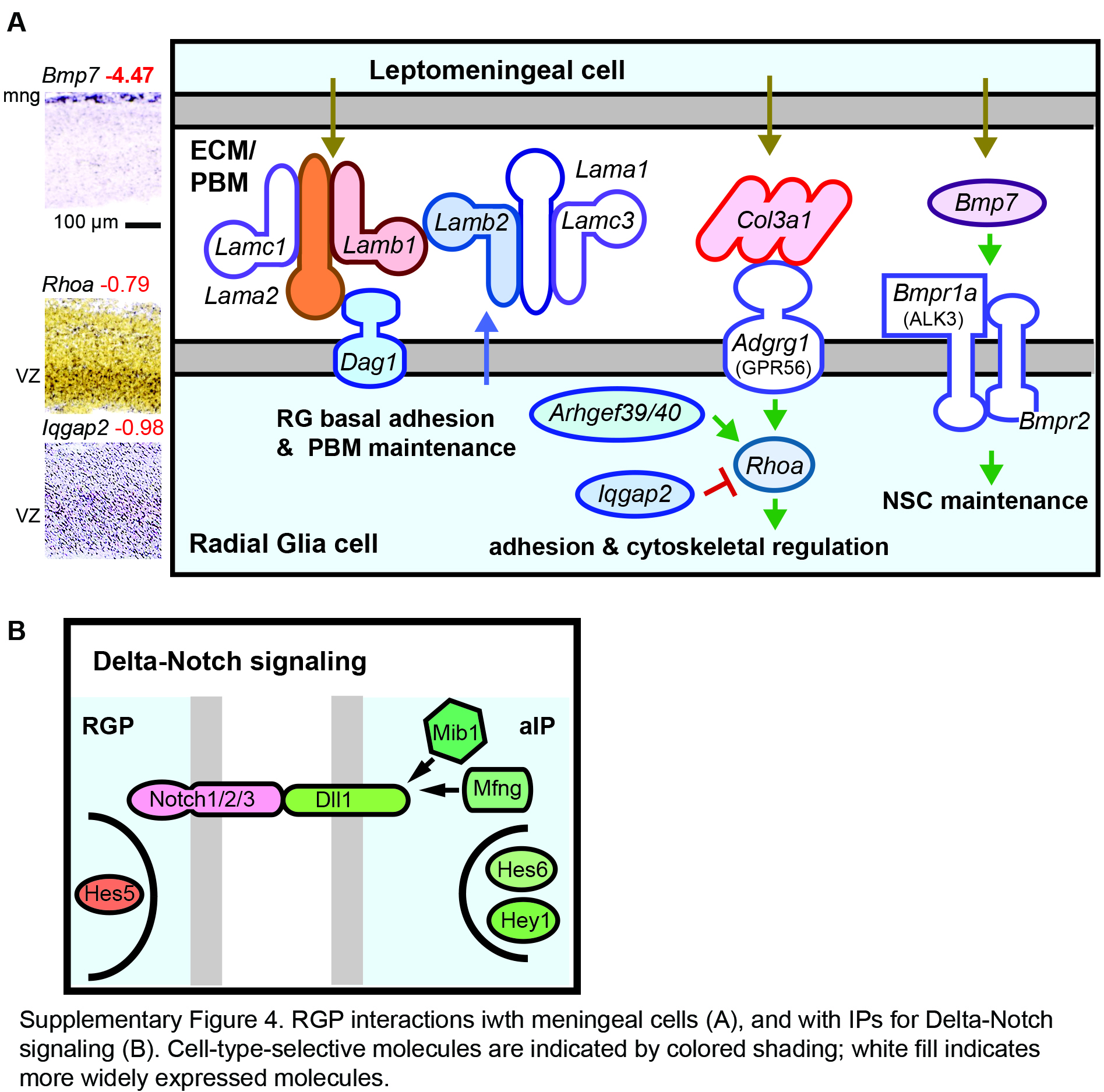

Supplement: Supplementary file 4 [file Image_4.JPEG]

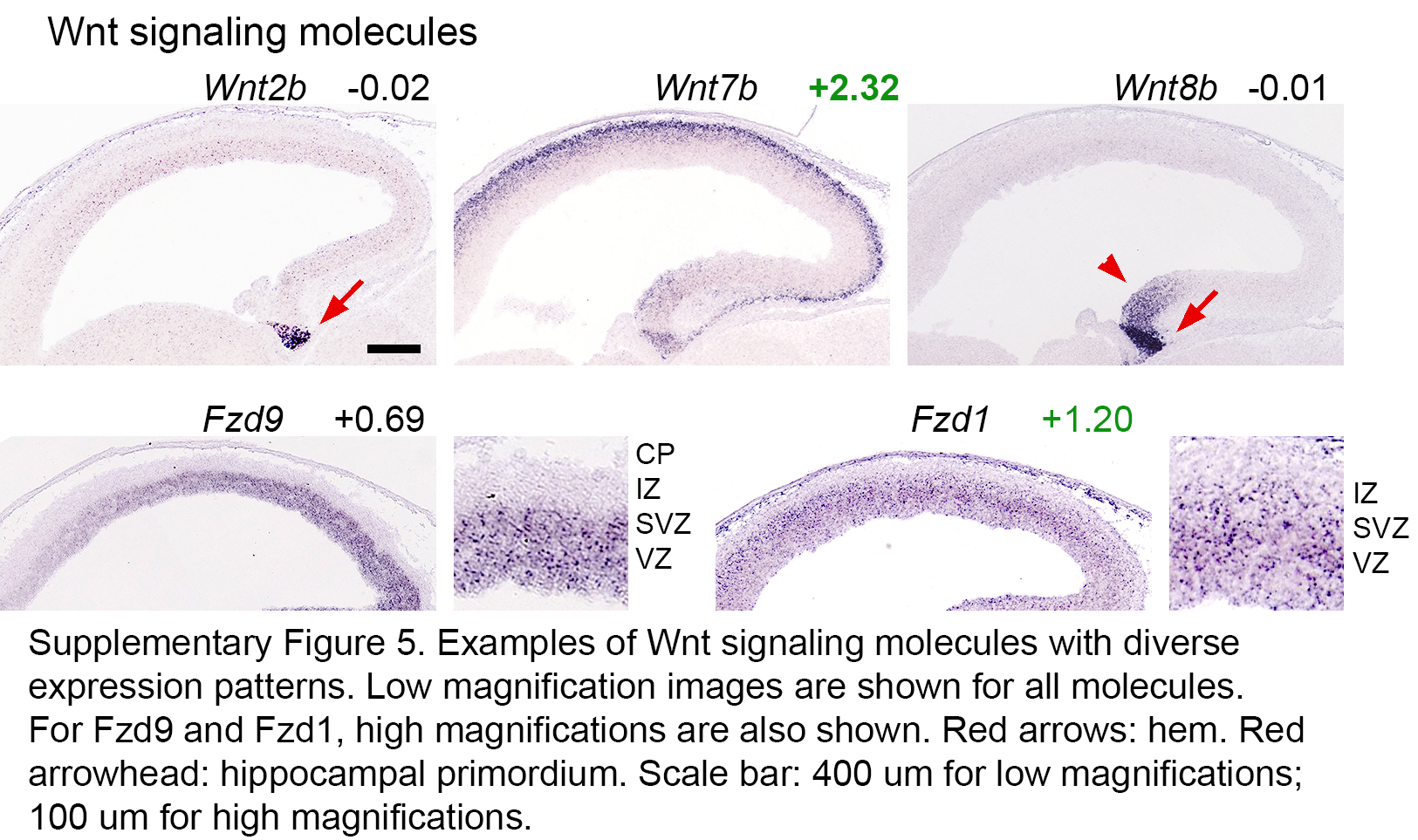

Supplement: Supplementary file 5 [file Image_5.JPEG]
